# Molecular dynamics simulations reveal the hidden EF-hand of EF-SAM as a possible key thermal sensor for STIM1 activation by temperature

**DOI:** 10.1016/j.jbc.2023.104970

**Published:** 2023-06-27

**Authors:** Andrei Neamtu, Dragomir N. Serban, Greg J. Barritt, Dragos Lucian Isac, Tudor Vasiliu, Aatto Laaksonen, Ionela Lacramioara Serban

**Affiliations:** 1 Department of Physiology, “Grigore T. Popa” University of Medicine and Pharmacy, Iasi, Romania; 2 Center of Advanced Research in Bionanocojugates and Biopolymers, “Petru Poni” Institute of Macromolecular Chemistry Iasi, Iasi, Romania; 3 Discipline of Medical Biochemistry, College of Medicine and Public Health, Flinders University, Adelaide, SA, Australia; 4 Department of Materials and Environmental Chemistry, Arrhenius Laboratory, Stockholm University, Stockholm, Sweden; 5 Centre of Advanced Research in Bionanoconjugates and Biopolymers, Petru Poni Institute of Macromolecular Chemistry, Iasi, Romania; 6 State Key Laboratory of Materials-Oriented and Chemical Engineering, Nanjing Tech University, Nanjing, P. R. China

**Keywords:** calcium, stromal interaction molecule 1 (STIM1), calcium release-activated calcium channel protein 1 (Orai1), molecular dynamics, endoplasmic reticulum (ER)

## Abstract

Intracellular calcium signaling is essential for many cellular processes, including store-operated Ca^2+^ entry (SOCE), which is initiated by stromal interaction molecule 1 (STIM1) detecting endoplasmic reticulum (ER) Ca^2+^ depletion. STIM1 is also activated by temperature independent of ER Ca^2+^ depletion. Here we provide evidence, from advanced molecular dynamics simulations, that EF-SAM may act as a true temperature sensor for STIM1, with the prompt and extended unfolding of the hidden EF-hand subdomain (hEF) even at slightly elevated temperatures, exposing a highly conserved hydrophobic Phe108. Our study also suggests an interplay between Ca^2+^ and temperature sensing, as both, the canonical EF-hand subdomain (cEF) and the hidden EF-hand subdomain (hEF), exhibit much higher thermal stability in the Ca^2+^-loaded form compared to the Ca^2+^-free form. The SAM domain, surprisingly, displays high thermal stability compared to the EF-hands and may act as a stabilizer for the latter. We propose a modular architecture for the EF-hand-SAM domain of STIM1 composed of a thermal sensor (hEF), a Ca^2+^ sensor (cEF), and a stabilizing domain (SAM). Our findings provide important insights into the mechanism of temperature-dependent regulation of STIM1, which has broad implications for understanding the role of temperature in cellular physiology.

Store-operated Ca^2+^ entry (SOCE), for example, Ca^2+^ release activated Ca^2+^ influx (CRAC) or capacitative Ca^2+^ influx (1, 2), is a major signaling pathway in various cells and physiological or pathological processes. SOCE is physiologically initiated by Ca^2+^ release from the endoplasmic reticulum (ER). Stromal interaction molecule 1 (STIM1), a type 1 transmembrane protein in the ER membranes, plays a central role in SOCE, an intricate process involving the cooperation of multiple partners. The two main factors therein are STIM1 in the ER membrane and the Orai1 Ca^2+^ channel in the plasma membrane (PM) (3, 4, 5, 6, 7, 8). STIM1, activated by [Ca^2+^] decrease in the ER, initiates a series of events leading to direct interaction of STIM1 with Orai1 and/or transient receptor potential channel 1 (TRPC1) and formation of "Ca^2+^ release activated Ca^2+^ channels" (CRAC) that subserve SOCE (5, 6, 9, 10, 11, 12). The topological structure of STIM1 (685 amino acids) shows a domain organization reflecting its complex functionality (Fig. 1). The intraluminal EF-hand/SAM (EF-SAM) Ca^2+^ sensor is connected by the transmembrane (TM) region to the cytoplasmic domains. The latter presents (a) the coiled-coil domain, which includes the CAD/SOAR region that binds to and activates Orai1; (b) the serine-proline-rich (S/P) domain, important for the correct targeting of the STIM1 cluster to the ER-PM junctions; and (c) the lysine-rich polybasic (K) domain. The latter is involved in multiple interactions with regulatory factors (*e.g.* SARAF; SOCE-associated regulatory factor) (13, 14), with the N terminus of Orai1 (15), and with phosphoinositides in the plasma membrane (16, 17).

**Figure 1:**
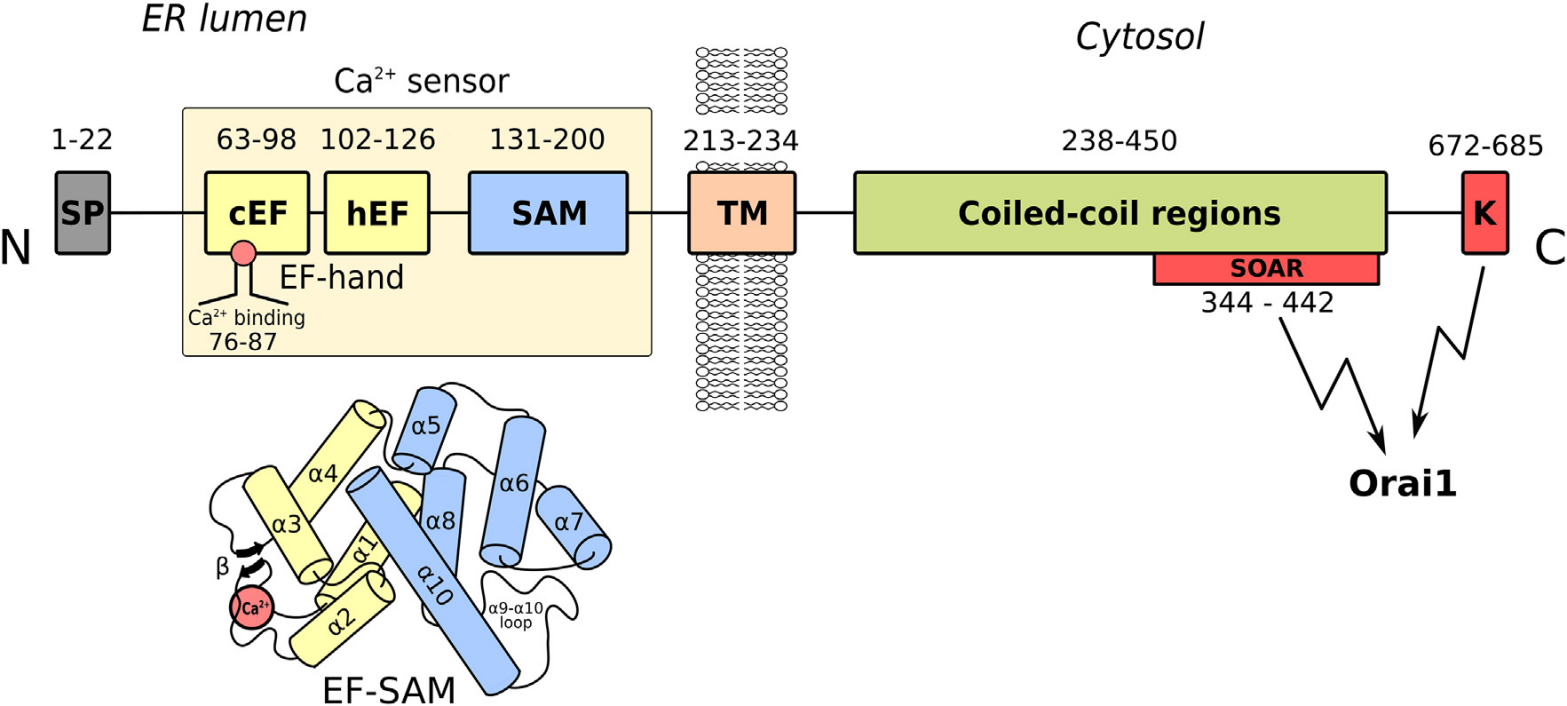
**STIM1 topological structure, depicting functional relevant domains, from N- to C-terminus**. The 3D structure of the Ca^2+^ sensor highlights EF-hand and SAM subdomains and the Ca^2+^ binding site (the small α9 helix, which lies between the α8 and the α9-α10 loop is occluded by the α10 helix in the depicted 3D structure). CAD/SOAR, CRAC activation domain/STIM1 Orai activating region; CC1-CC2-CC3, coiled-coil regions; cEF and hEF, canonical and hidden EF-hand; K, lysine-rich polybasic domain; SAM, sterile α-motif; SP, signal peptide; S/P, serine-proline rich domains; TM, transmembrane helix.

Unlike Ca^2+^ sensors responding to changes in cytosolic [Ca^2+^], in the range 10 to 1000 nM, stromal interaction molecules have evolved for sensing [Ca^2+^] changes in the ER, in the millimolar to micromolar range (18), by using the EF-SAM machinery. The luminal domain of STIM1 contains the Ca^2+^ sensor (Fig. 1), an EF-SAM complex made up of a sterile α motif (SAM) and two EF-hand motifs, that is, the Ca^2+^-binding canonical EF-hand (cEF) and the non-Ca^2+^-binding hidden EF-hand (hEF) (19, 20). The molecular model of STIM1 activation has been subjected to many recent reviews (7, 21, 22, 23, 24) and will not be detailed here. The current view describes the resting state of STIM1 as a dimer (25, 26, 27). In this state, EF-SAM domains are monomeric, with no contribution to STIM1 dimerization. A decrease in ER [Ca^2+^] triggers EF-SAM aggregation mainly through large-scale unfolding and exposure of hydrophobic residues (19, 20). EF-SAM dimerization forces molecular rearrangements of the STIM1 cytoplasmic domains, allowing STIMATE (STIM-activating enhancer) (28) to bind to CC1, followed by SOAR exposure and a robust STIM1 multimerization. Further translocation of multimeric STIM1 to ER-PM junctions (29) allows tethering and activation of Orai1 (15, 30, 31, 32, 33). Apart from these molecular rearrangements, other membrane constituents, like phosphoinositides and cholesterol, play important roles in SOCE (34).

STIM1 sensitivity to other physiological microenvironmental factors, such as oxidative stress (35), hypoxia (36), and temperature, represents another physiologically important aspect that is still awaiting better understanding. Earlier studies showed that STIM1 behaves as a thermal sensor, which is activated by a moderate increase in temperature independent of ER Ca^2+^ depletion (37). It appears that heat itself activates STIM1 and its accumulation to ER-PM junctions, while subsequent cooling provides coupling and activation of Orai1 channels ("heat-off" response), this last phase requiring the K-domain participation. But the hypothesized mechanism, involving EF-SAM unfolding as the initial trigger of STIM1 heat activation, could not be tested in the assays due to the usage of an EF-hand mutant (E87A) that constitutively forms "puncta" and mediates Ca^2+^ influx. Another study showed that SOCE in skeletal muscle cells is strongly regulated by temperature (38). Moreover, STIM1 can be activated by local heating induced by low-frequency magnetic fields (LFMF), triggering similar STIM1 translocation and Orai activation (39). *In vivo* experiments in skin keratinocytes presented STIM1 as a precise thermo-sensor: (a) clearly requiring its interaction with Orai1 and (b) an explanation for mammals’ ability to sense very small changes (<1 °C) from normal skin temperature (40). However, prior to Orai channels binding and crosslinking, STIM1 molecules must become activated first, in order to reach the ER-PM junctions. Thus, STIM1 could act as a novel type of primary thermosensor, with two distinct temperature-sensing sites: one that initially activates the resting-state STIM1 in the absence of ER Ca^2+^ depletion and another one that mediates Orai1 channels opening. This is a new concept in ion channels thermo-sensitivity where an intracellularly localized precursor, STIM1, becomes heat-activated and translocates to ER-PM junctions, subsequently leading to channel opening. The specific partner through which STIM1 interacts also impacts the thermal response, allowing for diversification depending on the context. For example, in the warm temperature range, the STIM1-Orai1 pair show a heat-off response while the STIM1-Orai3 pair displays a heat-on response (40).

Despite recent progress in the conformational dynamics of STIM1 activation, some issues remain unsolved. Hydrophobic-driven dimerization is now at the core of the proposed mechanism for triggering STIM1 activation. Still, there is no detailed mechanistic description of (a) the luminal EF-SAM loss of structure and exposure of hydrophobic core residues in low Ca^2+^ environments and (b) heat ability to activate STIM1. More puzzling and opposed to previous experimental evidence, recent far-UV circular dichroism spectroscopy and FRET measurements (41) indicated that large-scale unfolding of the EF-SAM domain is not necessary to induce STIM1 activation. These aspects remain controversial in the absence of a fully characterized Ca^2+^-depleted or heat-activated EF-SAM structure.

Molecular dynamics (MD) simulations constitute a well-established method to examine atomistic aspects of protein dynamics. MD simulations have been successfully used in the past to understand the mechanisms underlying the functions of other Ca^2+^-binding proteins, such as calmodulin (42, 43, 44), calbindin D9k (43, 45), parvalbumin (46), and troponin (47). Many of these studies focused on the relationship between Ca^2+^ binding/un-binding and the structural and/or conformational flexibility of the analyzed proteins, this way complementing static structural information available from experiments. The mechanistic of Orai channel activation and gating have been extensively studied by molecular dynamics simulations (48, 49, 50, 51, 52). However, to our knowledge, there are only four MD studies of EF-SAM available to date. In the first study of Furukawa *et al*. (53), the authors present the results of replica exchange MD (REMD) simulations, suggesting that unfolding involves a Ca^2+^-free intermediate with structure loss in the α9-α10 region of SAM. However, due to the limited time scale (tenths of nanoseconds), the results did not allow for a more detailed description of EF-hands involvement therein, or for an explanation of how the hydrophobic interface between EF-hands and SAM is affected by Ca^2+^ un-binding. In the second study, Mukherjee *et al*. (54) used conventional MD calculations to capture the early conformational changes involved in EF-SAM dimerization and to identify the key residues associated with this process. Notably, this study evaluates the EF-SAM dynamics in its native membrane-anchored environment. Although many interesting results emerged, the calculations were performed at a fixed temperature of 27°C, which hinders predictions on the temperature dependence of EF-SAM activation. The two other studies by Schober *et al*. (55) and Sallinger *et al*. (56) used a combined experimental and MD approach to gain more detailed insights into Ca^2+^ sensing and sequential activation of STIM1. They identified, using flooding simulations (*i.e.* performed in extremely high Ca^2+^ concentration), residues in the cEF and hEF domains of STIM1 that can bind multiple Ca^2+^ ions. However, they also found that a single Ca^2+^ ion is sufficient to stabilize the luminal domain. Furthermore, their MD simulations revealed that the F108I mutation destabilizes the hEF hand on a timescale of hundreds of nanoseconds. While all these studies did not directly address the role of temperature in STIM1 activation, they provide valuable insights into the structural dynamics of EF-SAM and its response to mutations.

Here we used conventional MD and enhanced sampling MD to investigate molecular mechanisms of temperature sensing by STIM1 and the crosstalk between temperature- and Ca^2+^-sensing. The conventional MD simulations at different temperatures, together with the replica exchange with solute tempering (REST2) simulations, offered a possible mechanistic explanation for STIM1 temperature sensing and its decoupling from Ca^2+^ sensing. The results obtained helped us to design a functional model of the EF-SAM sensor, with the following specific roles of the subdomains: hEF-SAM is the temperature sensor, cEF-hand is the [Ca^2+^] sensor, and SAM is a stabilizer for both sensors in the inactive state of STIM1.

## Results

### Strategy to investigate conformational changes upon heating and/or Ca^2+^ removal

In view of a mechanistic explanation for the thermal sensitivity of STIM1, we examined the conformational changes in the EF-SAM domain at different temperatures. The interplay between Ca^2+^ and thermal sensing of STIM1 was also addressed. Probing for the inceptive structural rearrangements induced by Ca^2+^ dissociation at different temperatures gave us valuable insights on the molecular “hot-spots” critical for the activation process. Thus, we first used conventional MD simulations (up to the microsecond time scale), followed by enhanced sampling replica exchange with solute tempering (REST2) simulations, the latter being necessary to describe the large-scale conformational changes of EF-SAM in response to the studied stimuli (*i.e.* Ca^2+^ depletion and/or heating).

**Figure 2:**
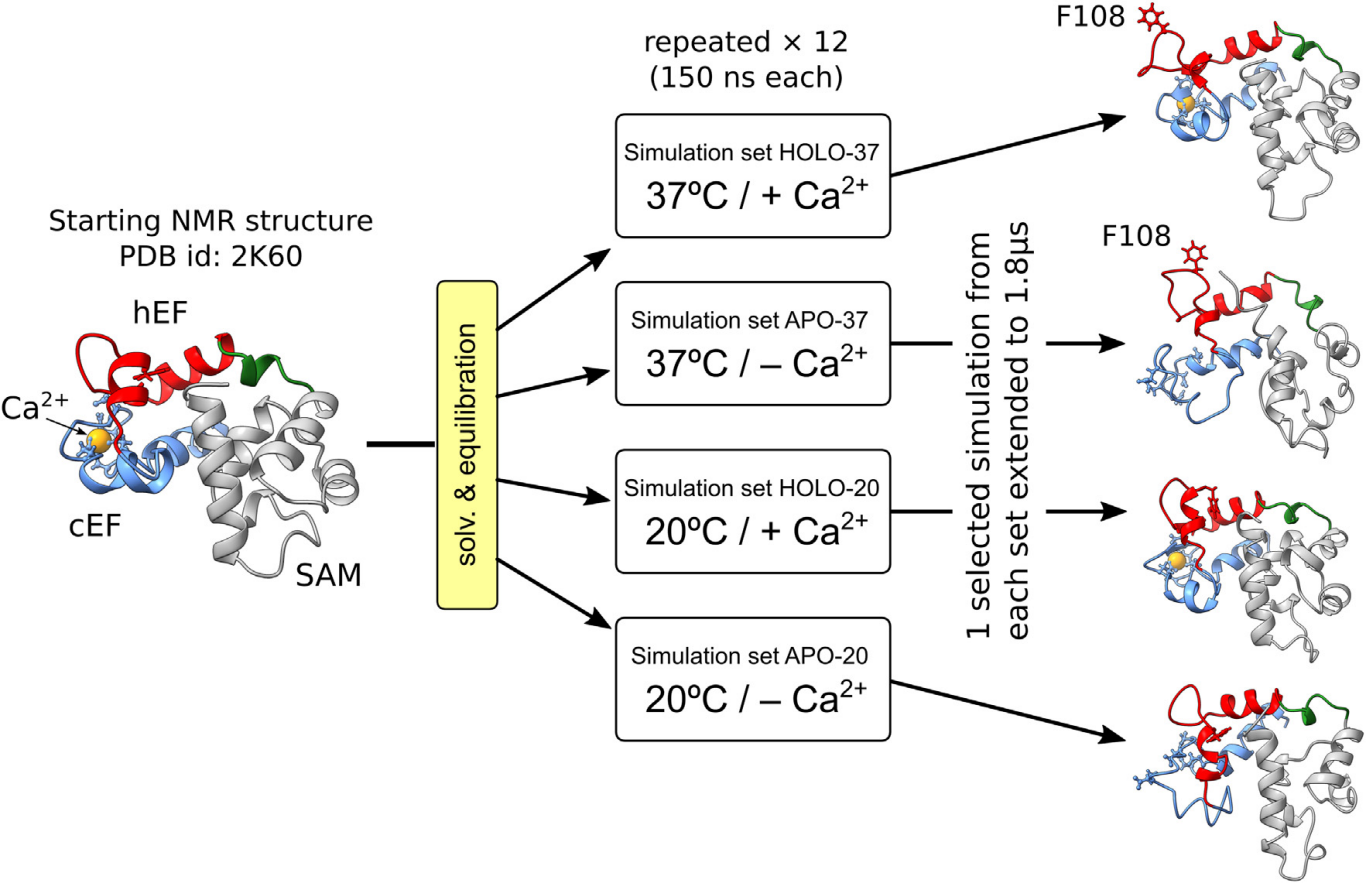
**General workflow for the conventional molecular dynamics simulations of the EF-SAM domain of stromal interaction molecule 1 (STIM1).** The starting structure was based on the NMR model (2K60) of the Ca2+-bound EF-SAM of STIM1 at 20 °C. Simulations were performed at two temperatures, 20 °C and 37 °C, with and without bound Ca2+. Each set consisted of 12 repeated simulations, and one simulation from each set was extended to 1.8 μs. The cEF (*blue*), hEF (*red*), and SAM (*grey*) subdomains were color-coded for clarity, with the small α5 helix connecting EF-hand to SAM subdomains shown in *green*. The residues involved in Ca2+ binding sites (*blue*) and the Phe108 residues (*red*) were depicted as *sticks*.

The overall simulation approach for the conventional MD simulations is schematized in Figure 2. Additionally, Fig. S5 provides a visual representation illustrating the spatial arrangement of the relevant residues discussed in this article within the overall structure of EF-SAM. To assess the structural effects of Ca^2+^ removal, two types of MD simulations were initially considered. The first type examined the EF-SAM domain with Ca^2+^ removed (APO-37) and the second one considered the cEF site occupied by one Ca^2+^ cation (HOLO-37), the latter being used as reference. Both simulation types were performed at 37 °C to mimic physiological conditions. However, as preliminary simulations evolved, two facts became evident. First, at 37 °C with Ca^2+^, the RMSD of the protein backbone displayed large deviations from the NMR structure over the first several hundred nanoseconds, analogous to those recorded for the Ca^2+^ free form. Deviations were as high as 6 Å, reflecting large conformational drifts in both cases. This could be anticipated for the Ca^2+^ free form, but not for the Ca^2+^ bound form and it was attributed to the temperature difference between the simulations (37 °C) and the NMR experiment (20 °C) (20). In general, temperature-driven conformational drifts are expected for a heated protein. Given the proven thermal sensitivity of STIM1, these preliminary data raised the question of whether the EF-SAM may have the peculiar thermal properties of a thermal sensor as suggested previously (37) but not clarified yet. Also, a profound conformational alteration of cEF in the Ca^2+^ binding site loop developed early (50–150 ns) in the Ca^2+^-free form. While the Ca^2+^ binding site expansion is an expected result of Ca^2+^ removal in EF-hand proteins, the extensive unfolding of the EF-SAM binding loop is atypical although previously mentioned for some EF-hand proteins (57, 58). Thus, the unconventional structure of EF-SAM, which pairs together a Ca^2+^ sensitive EF-hand (cEF) and a Ca^2+^ insensitive one (hEF), may impose uncommon structural responses upon either Ca^2+^ loss or temperature rise or both.

Simulations at the NMR experiment temperature were thus required to further clarify such aspects. Therefore, four simulation conditions were used: T = 20 °C with Ca^2+^ (HOLO-20), T = 20 °C without Ca^2+^ (APO-20), T = 37 °C with Ca^2+^ (HOLO-37), and T= 37 °C without Ca^2+^ (APO-37). Initially, for each combination, 12 short individual simulations (150 ns) were performed, starting with different initial conditions (atom velocity distributions). This was necessary to test the reproducibility of the observed large binding loop expansion in the Ca^2+^ free form and its stability in the Ca^2+^ loaded form and how this is affected by temperature. To investigate longer time scale behavior, four simulations (one per each set of 12) were extended to 1.8 μs.

As we used for the metal center a non-bonded model, Ca^2+^ stability was carefully evaluated on the microsecond timescale. The results (Fig. S1) showed that the Ca^2+^ ion remained coordinated in a pentagonal bipyramidal geometry, in both HOLO-37 and HOLO-20; thus, these simulations can be considered valid to describe the EF-SAM dynamics in the Ca^2+^-loaded form.

The correlations in low-frequency atomic displacements (*i.e.* large conformational changes) are undersampled in conventional MD, even on the μs time scale. Therefore, we used REST2 to evaluate EF-SAM conformations at different reference temperatures and also the EF-SAM thermal unfolding curves in the presence and absence of Ca^2+^. The outcome of the latter could be directly compared with experimental data from the literature.

### Structural stability of the Ca^2+^ loaded form of EF-SAM at 20 °C and validation against experimental data

**Figure 3:**
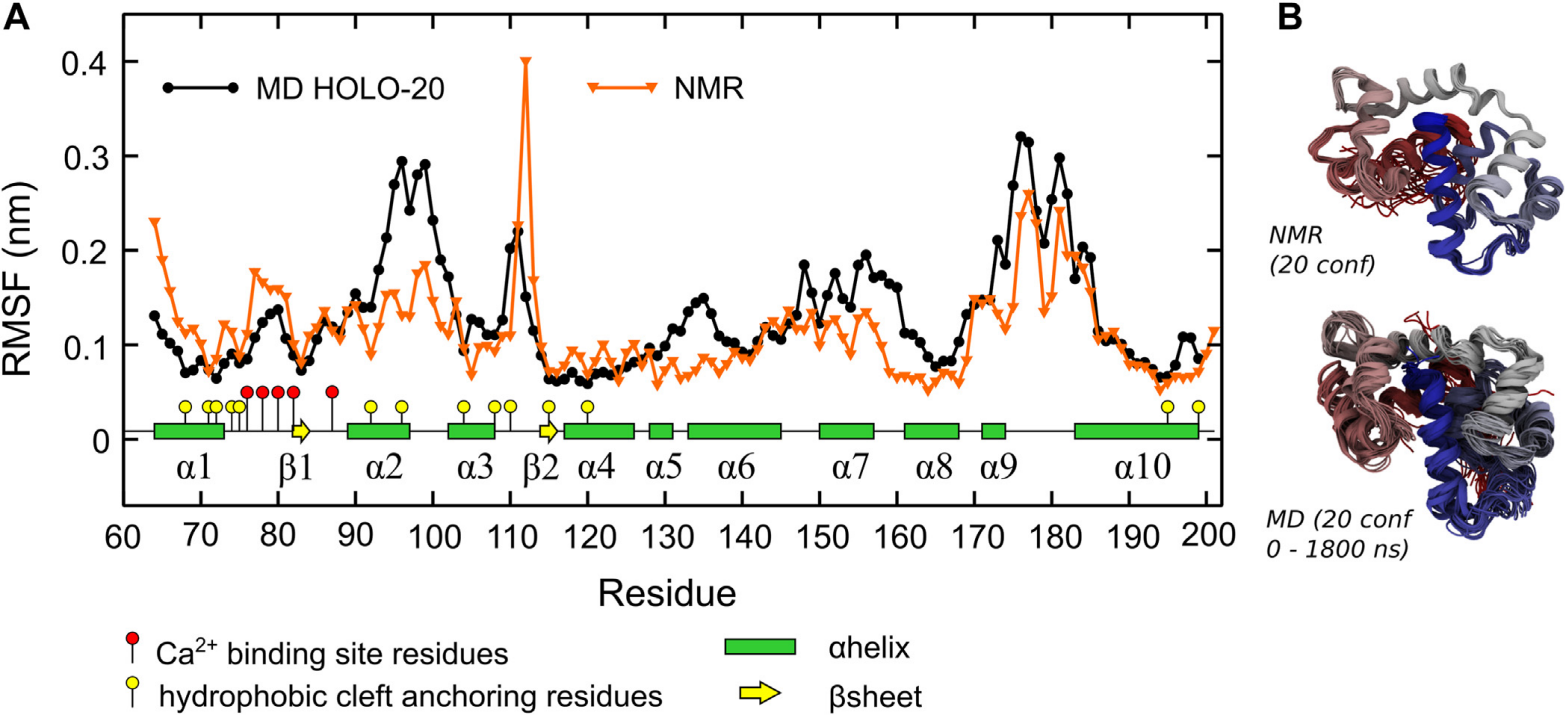
**Comparison of the molecular dynamics simulations with the available experimental data.***A*, root mean square fluctuations (RMSF) of atomic positions averaged over each residue for the extended simulation with Ca^2+^ (HOLO) at 20 °C (*black*); the RMSF of the NMR ensemble (20) (*orange*), resolved at 20 °C, was superimposed for direct comparison. Ca^2+^ binding site residues and hydrophobic cleft anchoring residues were highlighted by *red* and *yellow* "*ball* and *stick*" glyphs. *B*, ribbon diagrams of the 20 conformations in the NMR ensemble and of 20 equally spaced (90 ns) conformations from the MD trajectory, colored as a *rainbow* from *red* (N-t) to *blue* (C-t).

**Figure 5:**
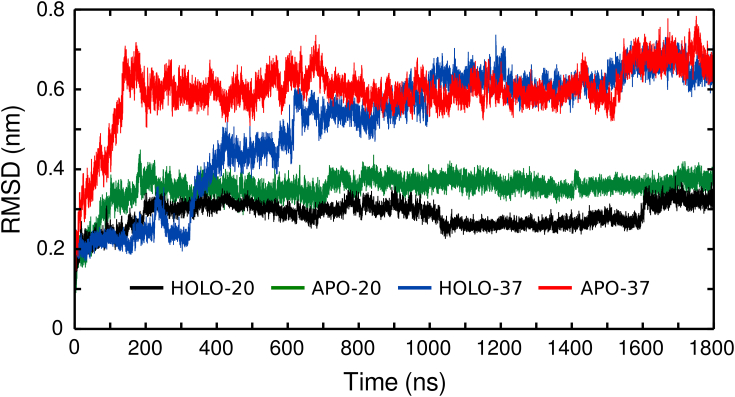
**Root mean square deviations (RMSD) of the EF-SAM C-α atoms relative to the starting experimental NMR structure, in presence of Ca**^**2+**^**at 20 °C (HOLO-20) and at 37 °C (HOLO-37) and without Ca**^**2+**^**at 37 °C (APO-37) and 20 °C (APO-20), respectively**.

EF-SAM had a well-preserved structure along the entire 1.8 μs of HOLO-20 simulation, with an average RMSD relative to the NMR structure lower than 0.35 (Fig. 5). For further comparison with the experimental data, we evaluated the root mean square fluctuations (RMSFs) of atom positions averaged over each residue (Fig. 3). Considering the good EF-SAM structure conservation, these RMSFs represent true fluctuations of atomic positions and not structural drifts. Figure 3*A* shows the MD RMSFs together with the fluctuations computed from the experimental NMR ensemble (20) (multiplied by an arbitrary constant, for easier qualitative comparison). The protein regions poorly defined in the NMR experiment also show large RMSF within the MD ensemble (Ala79-Asn80, Asp95-His99, Glu111-Asp112, Pro148-Leu157, Thr170-Ser185). The NMR structure of EF-SAM is a minimal construct of the N-t domain of STIM1, stable and folded in the presence of Ca^2+^, with an artificial sequence Gly(-5)-Ser(-4)-His(-3)-Met(-2)-Ala(-1)-Ser(0) added to its N-t Ser58. This sequence, together with Ser58-Lys62, was extremely flexible, lacking any defined secondary structure, in both the NMR and the MD ensembles, so they were not included in RMSF analysis. The least well-defined residue in the NMR ensemble is Asp112, belonging to the hEF α3-β2 loop, together with Glu111 and Lys113. The protein backbone displayed large fluctuations in this region in the MD ensemble too. Still, the Ca^2+^ binding site residues showed good stability in both the calculated and the experimental data. Hydrophobic cleft residues of the EF-hand subdomain (Val68, Ile71, , Leu74, Met75, Leu92, Phe108, Ile115, and Leu120) and α10 anchoring residues (Leu195, Leu199) also displayed comparable low fluctuations in both ensembles (MD and NMR). One exception was Leu96 in the C-t region of α2, with larger RMSF in the MD ensemble. Also, the α2-α3 loop and the N-t part of α6 were more flexible in MD trajectories *versus* their neighboring sequences, a feature that was not apparent in the NMR ensemble. Within the SAM domain, NMR and MD RMSF showed correlated increased flexibility of α6-α7 loop, α7 helix, and α9-α10 loop. Figure 3*B* shows a direct comparison between the MD and experimental NMR conformational ensembles. The secondary structure analysis, detailed in Fig. S2, also revealed good conservation of the structural elements during the simulation in Ca^2+^ presence at room temperature.

These results show that the chosen NMR structure and the force field used to describe the molecular models constitute a reasonable basis for modeling. Next, we used these models to investigate how Ca^2+^ removal affects EF-SAM stability at different temperatures.

### Effect of Ca^2+^ removal on the structural stability of EF-SAM at different temperatures

Here we discuss the structural impact of Ca^2+^ removal on the binding site of EF-SAM at 20 °C and 37 °C, respectively. The cEF coordinates Ca^2+^ in a pentagonal bipyramidal configuration. In the 2K60 NMR structure, the residues involved in Ca^2+^ binding include Asp76(1,X), Asp78(3,Y), Asn80(5,Z), Asp84(9,-X), and Glu87(12,-Z) (usual EF-hand notations for residue linear positions and alignment orientation). Position “9” is probably occupied by a water molecule, H_2_O (9,-X), while the Asp82(7,-Y) side chain has no contact with Ca^2+^ in the NMR structure.

**Figure 4:**
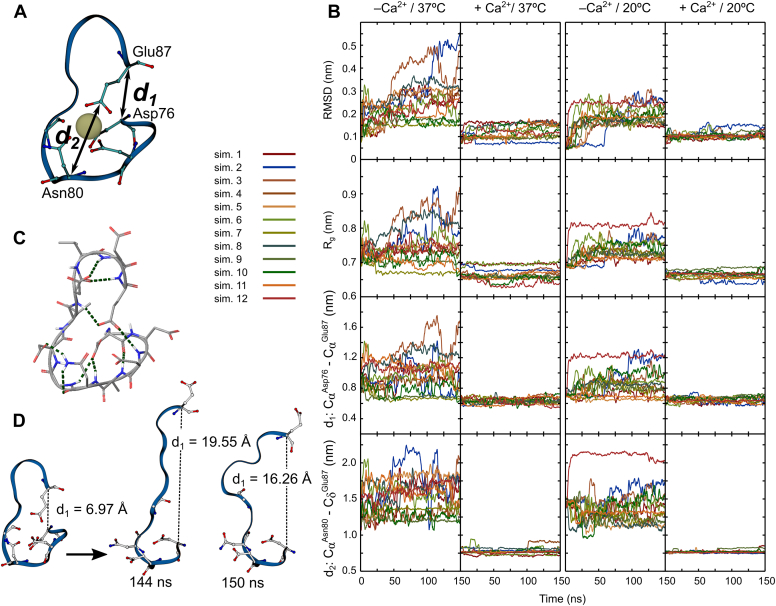
**Structural effects of Ca**^**2+**^**removal on the cEF at 20 °C and 37 °C.***A*, model of the Ca^2+^ binding loop with the chelating residues represented as sticks and the definition of the *d*_*1*_ and *d*_*2*_ distances. *B*, *top to bottom*: root-mean-square deviations (RMSD) of C-α carbon atoms; radius of gyration (*R*_*g*_); *d*_*1*_ and *d*_*2*_, at 37 °C and 20 °C, in the presence or absence of Ca^2+^, repectively. *C*, the hydrogen bonding network of the Ca^2+^ binding loop residues. *D*, conformational models of the Ca^2+^ binding loop recorded at 144 ns and 150 ns respectively in the largest RMSD APO-37 simulation (Sim.2, *blue line* in (*B*) graphs).

The initial effects of calcium removal could be evaluated from the repeated short-length MD simulations (150 ns each) (Fig. 4). A combination of four parameters was used to assess the stability of the cEF Ca^2+^ binding site loop (1): the α carbon RMSD (2); the radius of gyration *R*_*g*_ of the sequence Leu74 to Ser88 (3); the distance *d*_*1*_ between C-α of Asp76 and C-α of Glu87 (4); and the distance *d*_*2*_ between C-α of Asn80 and C-δ of Glu87 (Fig. 4*A*). All four parameters showed larger deviations for the APO vs HOLO simulations at both 20°C (smaller differences) and 37°C (larger differences; RMSD increased even up to 5 Å in APO-37 vs only 2 Å in HOLO-37). The *R*_*g*_ showed Ca^2+^-dependent variations, larger for the Ca^2+^-free forms. These variations correlated well with the increased RMSD values in the same conditions. The larger values of C-α RMSD indicate that Ca^2+^ removal induces backbone destabilization, while elevated *R*_*g*_ suggests an expansion of the binding pocket and an increased side chain disorder, as confirmed by visual inspection of the MD trajectories. Distances *d*_*1*_ and *d*_*2*_ gave a more detailed picture of the local rearrangements following Ca^2+^ removal. The entering and exiting α helices within cEF (α1 and α2 respectively) come into contact through Asp76 and Glu87, residues of the Ca^2+^ binding pocket. The distance *d*_*1*_ between the C-α of these residues was considered a measure of the binding pocket expansion. There are significant differences between *d*_*1*_ measured in APO vs. HOLO, up to *d*_*1 APO*_ *– d*_*1 HOLO*_ ≈ 12 Å in some of the simulations (Fig. 4, *B* and *D*). Thus, the removal of Ca^2+^ was shortly followed by marked expansion of the Ca^2+^ binding pocket at both 37 °C and 20 °C.

The bidentate side chain ligands of the 12th loop residue (Glu87) are known to be of critical importance to the EF-loop structure stability. Despite the Glu87 location at three residues away from the last chelating amino acid, the side chain of Glu87 strongly interacts with the cation, facing toward the rest of the chelating residues. The distance *d*_*2*_, between the C-δ of Glu87 and the C-α of Asn80 was chosen as a metric of preservation or loss of this orientation. As seen in Figure 4*B*, the *d*_*2*_ values are higher for APO than for HOLO both at 37 °C and at 20 °C. This is due to Glu87 side-chain reorientation in the absence of Ca^2+^, in a position facing away from the binding site. Simulations displaying highly increased *d*_*2*_ also showed increased *d*_*1*_ values (*i.e.* BLE). In contrast, the Ca^2+^ binding loop showed good structural stability in the HOLO forms, at both 37 °C and 20 °C, with the conservation of all analyzed structural parameters.

Such BLE on short time scales induced profound EF-SAM destabilization on extended time MD (μs). Several hydrophobic interactions stabilize the well-folded helical structure in the Ca^2+^-loaded form. Each EF-hand has a core of hydrophobic residues between the entering and exiting helices, including Met75, Val83, Val85, Leu92, and Leu96 for cEF and Phe108, Leu114, and Ile115 for hEF, respectively. The EF-pairing is also stabilized by these residues, as they come into close contact in the resting folded state (Fig. S3*A*). Three hydrophobic residues, strategically placed on the α10 helix of SAM (namely, Leu192, Leu195, and Leu199), anchor the α10 helix inside the hydrophobic cleft provided by the EF-hand pair. These interfaces allow for “tripartite” cEF-hEF-SAM hydrophobic interactions hat stabilize the compact, well-folded conformation of EF-SAM. The stability of the inter-EF hand (Fig. S3*B*) and EF pair–SAM (Fig. S3*C*) hydrophobic interfaces was monitored by constructing inter-residue hydrophobic contact maps at different times during APO and HOLO simulations. Both the cEF–hEF interface and the EF pair–SAM interface were undermined by Ca^2+^ removal, with many direct contacts between the above-mentioned hydrophobic residues being lost (as detailed in Figs. S3 and S4). A certain degree of hydrophobic destabilization occurs in the presence of the Ca^2+^ ion at 37 °C vs 20 °C, although the general architecture of the “tripartite” hydrophobic interface is preserved. Most notably, at 37 °C Phe108 in the hEF loses its contact with Leu120, Ile115, and Leu199 due to transient exposure of its sidechain towards the solvent; this does not happen at lower temperatures, as discussed below. Given the conformational alterations observed at 37 °C, especially for the Ca^2+^ loaded form, we then explored the local impact of temperature on different EF-SAM regions.

### Partial unfolding of EF-SAM in the Ca^2+^ loaded form at near-physiological temperatures is present in hEF but not in cEF

The time evolution of RMSD for the Cα atoms of EF-SAM in HOLO-37 displayed large variations, reflecting important conformational drifts from the experimental structure (Fig. 5). After the first ∼150 ns, when the protein remained conformationally stable, the RMSD rapidly increased up to 0.7 nm. This transition was attributed to the different temperatures of the simulation (37 °C) compared with the NMR experiment (20 °C), as discussed above.

More insights were provided by the fluctuations of individual residues of the protein chain. As expected, RMSF analysis showed higher spatial fluctuations for HOLO-37 vs HOLO-20 (Fig. S6, *A* and *B*). Our simulations revealed that certain regions of the EF-SAM domain displayed higher RMSF values, indicating that local structural changes occurred due to increased temperature. Given that STIM1 can be activated by temperature even in the presence of Ca2+, it was important to carefully evaluate the temperature-induced conformational changes of EF-SAM in more detail. The most flexible fragment was Thr107-Phe108-His109-Gly110. The high local flexibility of α3-β2 could be related to its observed solvent exposure. The solvent-accessible surface area (SASA) of two representative residues from this region, Phe108 and Gly110, was plotted against time (Fig. S6*C*). These two residues were postulated to play an important role in the Ca^2+^ sensor function, because replacing Phe108 and Gly110 with Asn in EF-SAM mutants rendered the protein constitutively (±Ca^2+^) aggregated (20). SASA of Phe108 in fully extended Gly-Phe-Gly tripeptides has been previously established (∼2.2 nm^2^) (59). Based on this and the visual inspection of trajectories, we considered that Phe108 SASA values may indicate the following states: fully exposed (>1 nm^2^); surface adsorbed (0.25–1 nm^2^); buried (<0.25 nm^2^). Along the 1.8 μs of simulation, the side chain of Phe108 displayed little or no solvent exposure in HOLO-20. In contrast, in HOLO-37, Phe108 stays with its phenyl group lying at the protein surface for the initial ∼1.2 μs and then it fully exposes its side chain to the solvent in the last 0.6 μs. Gly110 also displays lower backbone SASA at 20 °C vs 37 °C.

**Figure 6:**
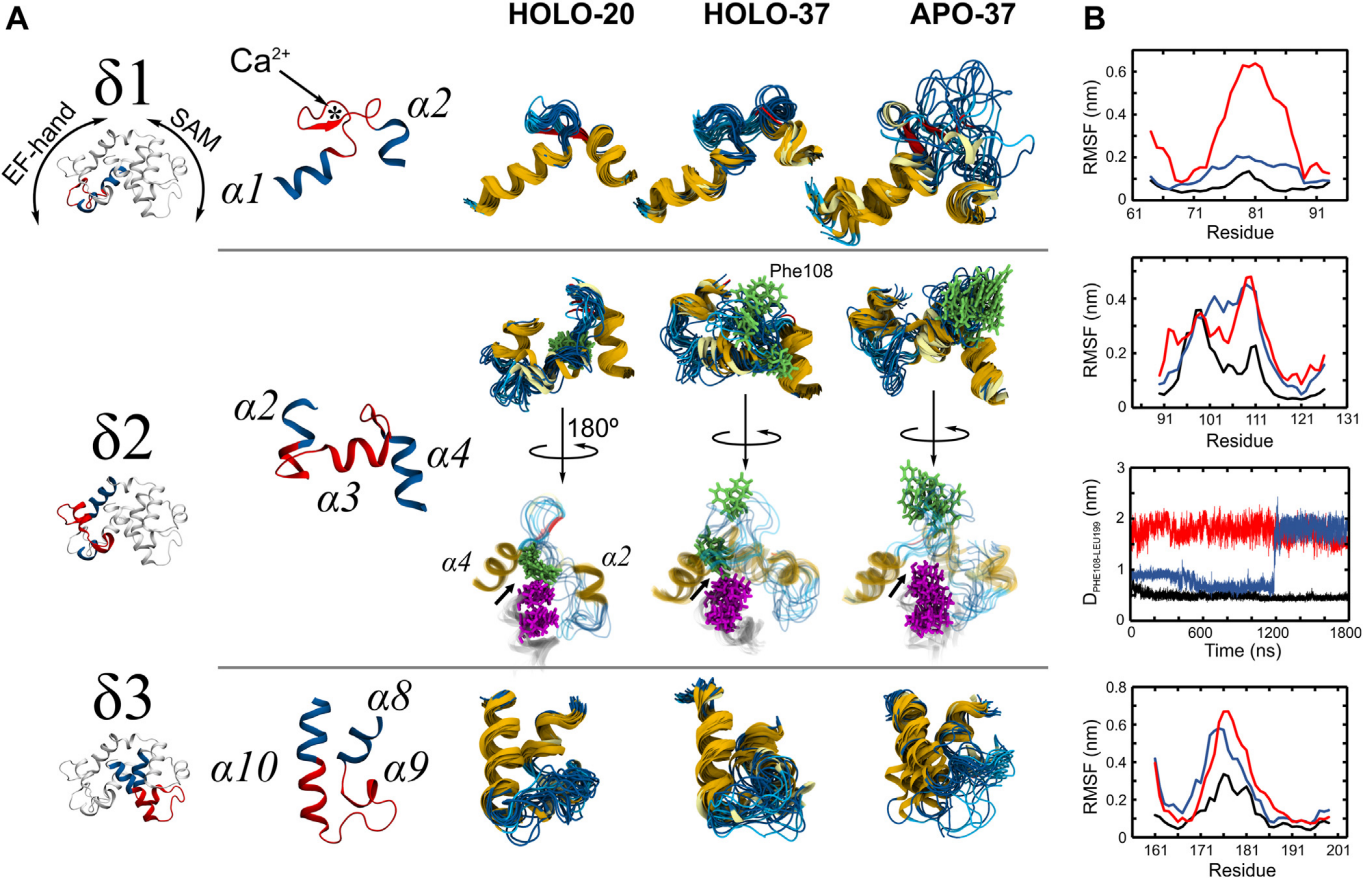
**Molecular dynamics (MD) simulations of the EF-SAM domain of stromal interaction molecule 1 (STIM1), with Ca**^**2+**^**bound (HOLO) or not (APO), at 20 °C or 37 °C.***A*, the three regions identified as prone to destabilization by Ca^2+^ removal (δ1, δ2, and δ3) and temperature increase (δ2 and δ3); in the *left panel*, the highly destabilized sequences are highlighted in *red*. In the *right pane*l, 10 equally spaced conformations extracted from the MD trajectories were over-imposed by fitting their stable endings (highlighted in *blue*). Phe108 is shown as *green sticks* (*arrow*), Leu199, and Leu195 are shown in *magenta*. The *arrow* indicates the interface between Leu199 and Phe108. *B*, RMSF of the δ1, δ2, δ3 residues for HOLO-20 (*black*), HOLO-37 (*blue*) and APO-37 (*red*). The distances between Phe108 and Leu199 C-α atoms were also plotted for the δ2 region (HOLO-20 in *black*, HOLO-37 in *blue*, APO-37 in *red*).

The above-discussed RMSFs were calculated for the entire protein and thus give information about the overall fluctuations of the polypeptide chain. However, due to large structural drifts in the HOLO and APO forms at 37 °C, globally fitting the protein backbone may hinder functionally important local fluctuations. These include fluctuations of polypeptide chain segments between conserved secondary structure elements. Based on global RMSF data and on secondary structure plots for HOLO-20 (Fig. S2), several most stable helices were selected for the separate local fitting of the MD trajectories, while the sequences between them were comparatively evaluated for increased flexibility and/or disorder. Three regions were identified as important for Ca^2+^ and temperature sensitivity (Fig. 6): (δ1) α1-α2 (residues 73–88) (cEF); (δ2) α2-α4 (residues 95–115) (hEF); (δ3) α9 and α10 (residues 171–184). The first region (δ1 in Fig. 6*A*), located in the cEF, corresponds to the Ca^2+^ binding site and is highly stable in both HOLO-20 and HOLO-37, showing no temperature-dependent instabilities. However, it becomes highly disordered in APO-37, as shown by much higher locally fitted RMSFs (Fig. 6*B*).

The second region (δ2), located mainly in the hEF, corresponds to the EF-hands intervening loop α2-α3, the α3 helix, and the α3-α4 loop. This region has increased flexibility and disorder, accompanied by loss of α3 secondary structure in both HOLO-37 and APO-37. Phe108 is located in the highly flexible α3-α4 loop, and its solvent exposure disrupts the interaction with Leu199, one of the main anchoring residues on α10. As suggested by the visual inspection of MD snapshots, the data confirmed that the close contact between Phe108 and Leu199 is (Fig. 6*B*): constantly present in HOLO-20; partially compromised in HOLO-37; completely disrupted in APO-37, followed by Phe108 side chain solvent exposure. Not only the Phe108-Leu199 pair is destabilized, but consequently the Leu199-Leu195 contacts are also affected, as snapshots representing their side chains are more scattered in Figure 6*A*. Thus, the hEF of STIM1 is highly temperature sensitive. Moreover, hEF contains in the “X” position a highly conserved hydrophobic residue among STIM1 proteins (Phe108) (Fig. S7), instead of the highly invariable Asp acidic residue usually found at this position among other EF-hand Ca^2+^ binding proteins. This change makes the hEF incapable of binding Ca^2+^, but able to hydrophobically interact with a nearby STIM1 hEF if Phe108 becomes solvent exposed, all in a temperature-dependent manner. So, hEF may act as a thermal activation “hot-spot” of STIM1, as further supported by our enhanced sampling simulations.

The third region (δ3) is highly stable for HOLO-20, but at 37 °C, it becomes disordered even in the presence of Ca^2+^; without Ca^2+^ the disorder is significantly more extended (including N-t of α10), as proven by the locally fitted RMSFs. This is in line with previous findings (53) that identified the loss of structure of the α9 - α10 region of SAM as an intermediary state, along the unfolding pathway of EF-SAM when Ca^2+^ is lost.

Based on the results obtained so far by equilibrium MD at two fixed temperatures, we further extended our analysis to the full thermal unfolding profiles of EF-SAM ± Ca^2+^, obtained by enhanced sampling MD techniques.

### Enhanced sampling simulations reveal the thermal sensor nature of EF-SAM

It is well known that conventional MD simulations suffer from the “sampling problem”, that is, the system becomes trapped in local minima, where it can remain for long periods of time, hindering the efficient sampling of the phase space. Conventional MD is not well suited to study the folding and unfolding of large proteins, occurring along tenths of microseconds to seconds. Conventional MD is limited to the description of the initial steps of such long events. To explore the unfolding and the thermal behavior of EF-SAM on a wider time scale, we used replica exchange with solute tempering (REST2) simulations. REST2 uses a number of “N” copies of the simulated system, called “replicas”, which are all simulated in parallel. Each replica is simulated at the same reference temperature but has a modified Hamiltonian with the soft degrees of freedom (electrostatic, van der Waals and dihedral terms) scaled down by a factor λ. The modified Hamiltonian is applied only to a part of the system, namely, the studied protein. This allows for an efficient passage over the conformational energy barriers using a small number of replicas compared with the original parallel tempering method (60). At specified time intervals the neighboring replicas are exchanged, or not, based on the Metropolis acceptance criterion. This way, the replicas diffuse in the λ space and efficiently explore conformations of the solute. In principle, one will follow only the base replica (λ = 1) trajectory for which the thermodynamic is exact. This trajectory becomes enriched in conformations coming from the “warmer” replicas (λ < 1) due to replica exchanges. However, using the method introduced by Stirnemann and Sterpone (61), the information contained in all the replicas can be successfully used to recover the thermal stability of proteins containing tenths of residues (62). The λ parameters, which scale down the intra-protein interactions, can efficiently be transformed to effective temperatures, and thus each replica can be considered to evolve at a different temperature. The major drawback is that calculations are extremely computationally intensive so that for large proteins they can only be efficiently performed on massively parallel supercomputers.

**Figure 7:**
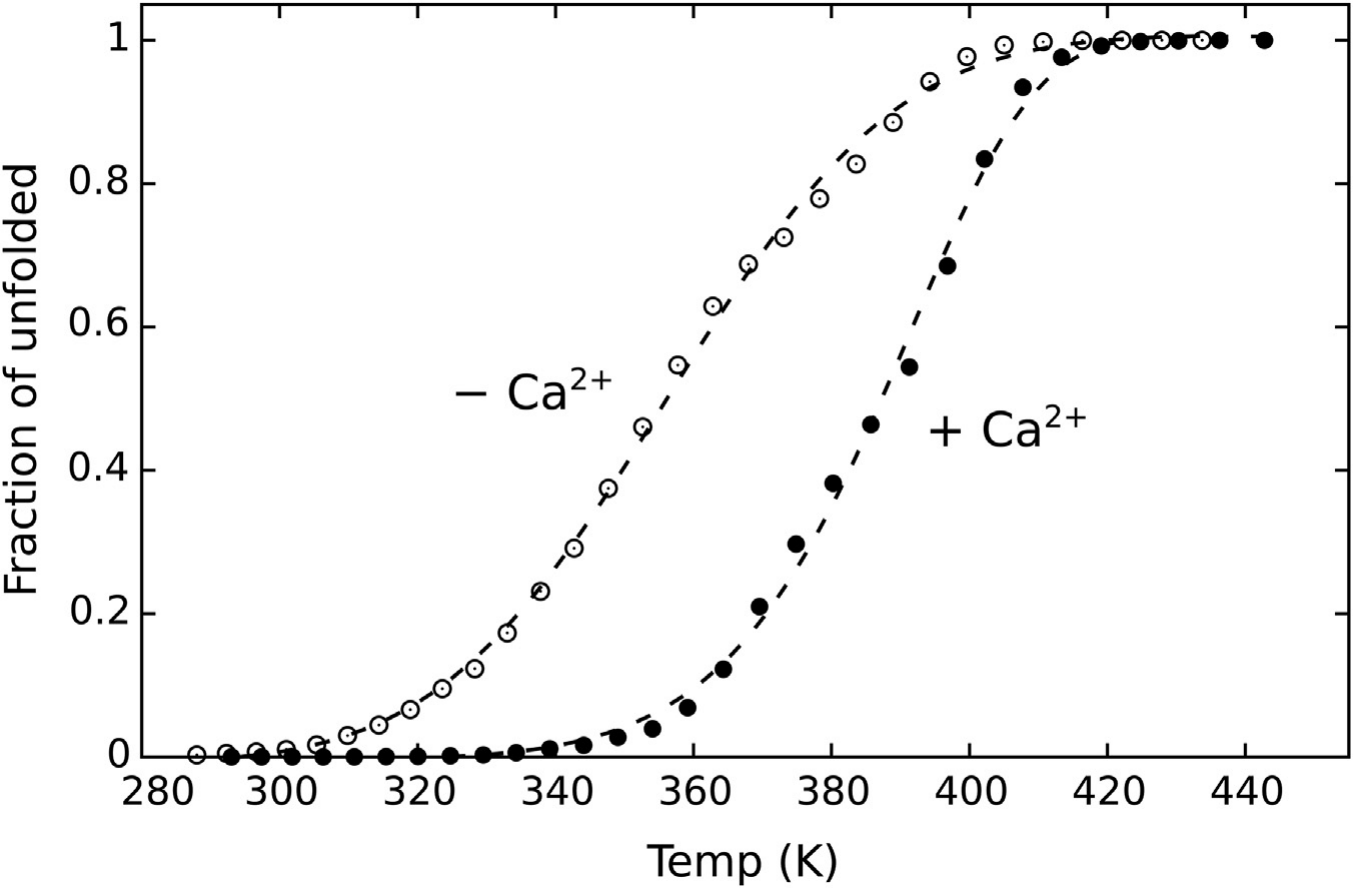
**Thermal stability curves of EF-SAM in HOLO and APO forms (+Ca**^**2+**^**and −Ca**^**2+**^**, respectively) obtained from the REST2 MD simulations**.

Based on the higher stability of the HOLO forms encounter in experiments and our conventional MD simulations, we have chosen the REST2 reference temperatures: 20 °C for HOLO and 15 °C for APO. The information contained in the “warmer” replicas (λ < 1) were used to construct the thermal unfolding profiles for the HOLO and APO forms. The Ca^2+^-free form of EF-SAM is much less stable than the Ca^2+^-loaded one, as shown by the unfolding profiles expressed as a fraction of protein helical content (Fig. 7). The shape of the calculated profiles and their relative place on the temperature scale correlates well with the thermal unfolding profiles experimentally determined by circular dichroism (19). Despite the potential overestimation of absolute temperature values in the REST2 simulations (61), the difference in the calculate melting temperature (T_m_) values between the Ca^2+^-free and Ca^2+^-loaded states was included as a single-parameter quantitative expression of the stability difference between the two unfolding curves, as the relative difference between the two states is likely to be less affected. The calculated value of 39 °C resembles well the experimentally measured value (26 ^o^C). As expected, Ca^2+^ had an important stabilizing effect on EF-SAM structure, maintaining the protein in a compact folded conformation at much higher temperatures vs the Ca^2+^ free form.

**Figure 8:**
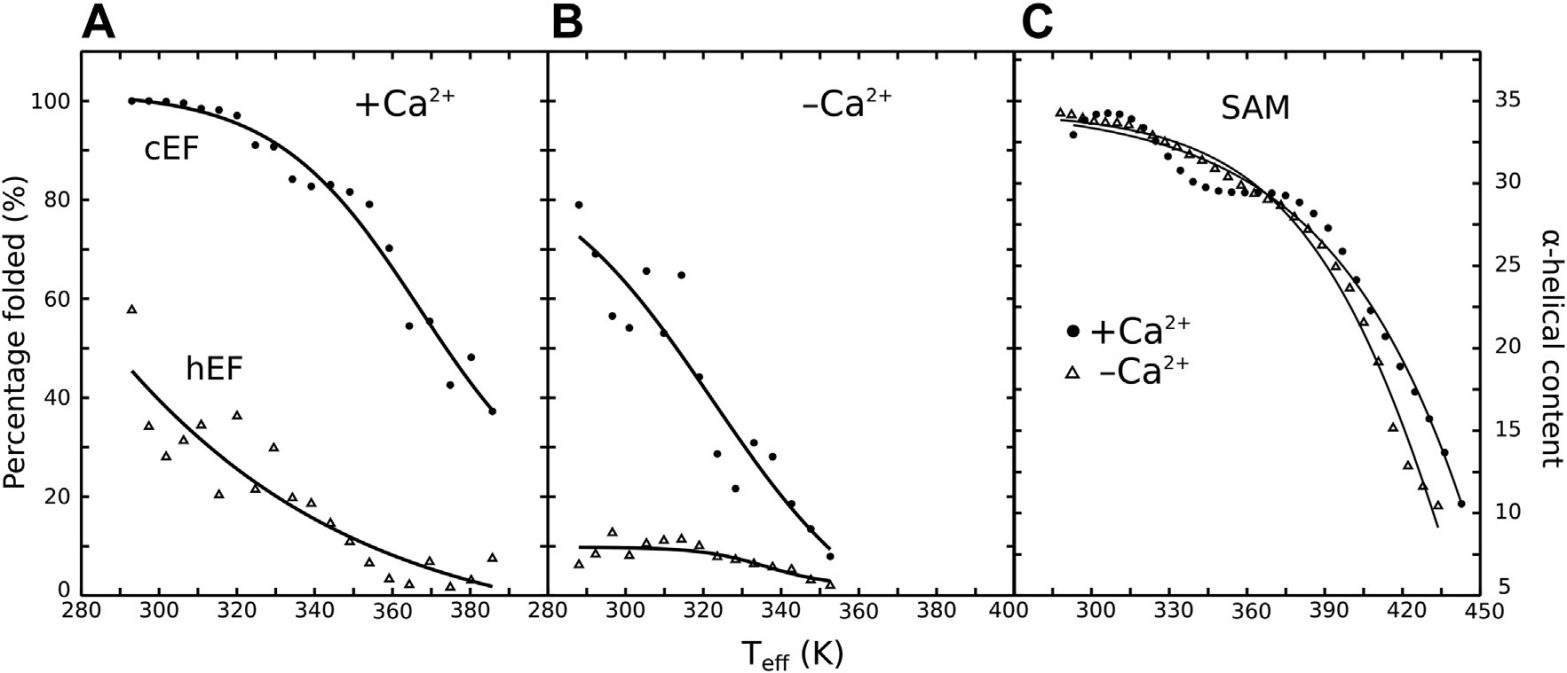
**Unfolding profiles of cEF, hEF, and SAM subdomains extracted from thermal unfolding simulations of the full-length EF-SAM protein in both HOLO and APO forms.***A*, EF hand in the Ca^2+^-loaded form, (*B*) EF hand in the Ca^2+^-free form, and (*C*) SAM in both Ca^2+^-loaded and Ca^2+^-free forms.

The profiles in Figure 7 give an overall estimation of the unfolding process for the entire EF-SAM domain. However, a separate evaluation of the percentage of folded states along the thermal unfolding profiles (see “Materials and methods” for the definition of the folded states) allowed us to split the global unfolding into individual contributions (Fig. 8). It should be noted that the unfolding profiles of cEF, hEF, and SAM domains depicted in Figure 8 are not individual thermal stability curves but rather the unfolding profiles of each subdomain within the context of the full EF-SAM protein. This approach was intentionally employed due to the highly cooperative nature of EF-SAM unfolding. By extracting the unfolding profiles from the simulations of the entire EF-SAM domain, we provide a convenient means to compare the thermal stability of individual subdomains and identify protein regions most susceptible to thermal perturbations. Upon examining the unfolding curves separately, one can observe that hEF, cEF, and SAM show different stability response curves with temperature rise. The cEF was much more stable than hEF, which readily unfolds even at moderate warming. In the HOLO form, cEF remains well folded even at 47 °C, while hEF becomes highly unfolded (Fig. 8*A*). As predicted by the conventional MD simulations, Ca^2+^ removal destabilized cEF, moving down its stability profile curve (Fig. 8*B*). However, analyzing the plots in Figure 8*B*, it is obvious that Ca^2+^ un-binding had a drastic effect on the overall structure of the EF-hand pair, compromising the stability of both cEF and hEF, even at low temperatures. This reflects the cooperative interactions between the two pairs of the EF-hand motif. On the other hand, SAM displayed high stability (Fig. 8*C*), maintaining high helical content even up to 80 °C in simulations.

## Discussion

Here we used advanced molecular dynamics simulations to gain insight into the thermal behavior of the intraluminal Ca^2+^ sensor of STIM1, which is known to be activated also by heat, and into the interplay between temperature sensing and Ca^2+^ sensing. The effect of heating on the EF-SAM Ca^2+^ sensor was evaluated at different temperatures, both in the resting state and after Ca^2+^ removal. The main results are summarized here. 1. We identified key residues and interaction interfaces that are important for the unfolding initiation. 2. There were large discrepancies in EF-SAM conformational preference at different temperatures in the resting state. These were not the overall effects of increased flexibility due to higher temperature. 3. Specifically, while the overall folding was preserved, the thermal-driven structural disorder appeared in the hEF-hand, which contains the hydrophobic Phe108, a highly conserved residue in this position among STIM1 proteins. Phe108 became solvent exposed in a temperature-dependent manner, an effect that promotes EF-SAM dimerization. 4. We identified Phe108 as a thermal “hot spot”. Using replica exchange simulations, we constructed in full atomistic detail the unfolding profiles of EF-SAM ±Ca^2+^, with results that strongly support the conventional MD findings. 5. We propose a modular architecture for EF-SAM, containing a Ca^2+^ sensor (cEF), a temperature sensor (hEF), and a stabilizing module (SAM).

Earlier evidence showed that, even at 37 °C, the STIM1 K-domain-deficient/Orai1 cells (*i.e.* not capable of the heat-response) show significantly lower basal Ca^2+^ levels and STIM1-dependent gene expression compared to the cells expressing the normal STIM1/Orai1. Wild-type cells instead showed heat-induced clustering of STIM1 at temperatures above 35 °C without depleting Ca^2+^ stores (37). This suggests that some "basal" levels of temperature-driven calcium influx, as triggered *via* STIM1, may be present at physiological temperatures, above but near 37 °C. Our conventional MD simulations indicate that the Ca^2+^ loaded EF-SAM displays temporary folded to unfolded transitions in the hEF-hand of STIM1 at 37 °C (Fig. S2), while simulations by Schober *et al*. (2019) using flooding simulations at the same temperature and high Ca^2+^ concentrations found hEF to be stable. This may be explained by the fact that the simulations of Schober *et al*. (2019) were performed in extremely high Ca2+ concentration (150 mM) to identify multiple Ca^2+^ binding sites, some of which were located in the hEF. Another reason may be related to the length of the simulations. An important unfolding event, consisting of Phe108 side chain exposure to solvent, occurred at around 1200 ns in our simulations (Fig. S6*C*), a time point that exceeds the length of the simulations presented in Schober *et al*. A direct comparison, however, must be regarded with caution as even on the microsecond time scale, the sampling of the conformational space is still probably insufficient. However, both studies found the SAM subdomain to be well-folded both in HOLO and APO forms, underlining the stability of this domain, which was also observed by us when assessing the thermal stability of EF-SAM. Our REST2 simulations further support our findings and provide a more comprehensive view on the hEF propensity to unfold at physiological and near-physiological temperatures. This may well explain the mentioned STIM1-involving behavior of temperature-responsive cells observed by others (37, 63, 64).

Based on circular dichroism experiments, it was previously proposed that loss of the secondary structure of EF-SAM reflects unfolding, accompanied by exposure of hydrophobic residues, followed by dimerization. But the low-resolution structural data in circular dichroism experiments did not allow for details about this process. The results of our REST2 approach bring full atomistic detail of the unfolding process and fully support the results that we obtained by conventional MD. The hEF and cEF unfolding profiles in Figure 8, *A* and *B*) show that the temperature responses of the two EF-hands are uncoupled. The cEF is stable in the physiological range of temperatures if Ca^2+^ is present, while the hEF presents partial disorder and prompt unfolding response to moderate temperature increase. Thus, the hEF may become unfolded due to moderate temperature rise, even in the presence of Ca^2+^, while the overall structure of EF-SAM is still preserved. The hEF unfolding is accompanied by the unmasking of the hydrophobic Phe108 side chain toward the solvent. So, this side chain is now unable to participate in the stabilizing “tripartite” hydrophobic interactions, while it becomes free to interact with other unfolded hEF-hands. This type of response indicates that hEF-hand is a possible temperature sensor within STIM1. Two findings strongly support this supposition (1): hEF is highly unstable thermally, compared to the rest of EF-SAM, in the physiological temperature range (2); hEF contains the highly conserved hydrophobic Phe108. Sequence alignments of hEF reveal that position 108 is persistently occupied by Phe residues in different known STIM1 proteins, while cEF motifs have a highly invariant Asp for Ca^2+^ binding in position 108 (Fig. S7). The invariance of the hydrophobic nature of the residues found in this position in STIM proteins unveils their relevance for the EF-SAM sensor functionality. Experimental and MD mutational studies, replacing Phe108 with Ile, showed a less thermally stable EF-SAM mutant (55) compared to the wild type, thus the Phe108 importance for EF-SAM stability. The thermal response of SAM, on the other hand, showed no dependence on EF-pair Ca^2+^ load and much higher temperature stability vs EF-pair Figure 8*C*). These results are consistent with current perspectives on cooperative protein folding/unfolding, which acknowledge: (1) the presence of significant dispersion in folding/unfolding rates across different protein structure sites, illustrating asynchronous structural changes occurring in multiple steps (65, 66) and (2) the tendency of small globular proteins to undergo folding/unfolding transitions predominantly populating only folded and unfolded states, effectively behaving as a single cooperative unit (67, 68).

The molecular mechanisms underlying temperature sensing in proteins are still a matter of debate. While the function of all macromolecules is altered by temperature, protein thermosensors provide information about the thermal environment due to their peculiar thermal properties, which allow them to trigger an appropriate physiological response upon heating or cooling. A particularly interesting example is the transient receptor potential vanilloid member 1 (TRPV1), a Ca^2+^-permeable cation channel involved in noxious heat and capsaicin sensing in humans (69). For a long time, it was unclear whether the heat sensing capability of TRPV1 is mediated by a dedicated modular heat sensor or by global conformational changes. A recent study (70), using cryo-electron microscopy to solve the TRPV1 structure at different temperatures, showed there is no distinct temperature sensor domain in TRPV1. Still, a subset of residues, around the outer pore and coupling domain, seems to be involved in the temperature sensing, further triggering stepwise conformational transitions up to channel opening. These residues form an interaction network directly involved in heat sensing. Notably, a single point mutation in this network, N628L, substantially decreases the heat sensitivity of TRPV1. This is an illustrative example of how a small, localized solvent exposure change of a residue may have a dramatic effect on the protein heat sensitivity. Several other studies demonstrate that changing the properties of a single residue may lead to significant effects on heat sensing (71, 72). Like in any other protein when heated, the EF-SAM domain of the thermosensor protein STIM1 suffers thermal-induced global effects on its structure. But, EF-SAM has a short strand of amino acids, i.e. hEF, that our data highlights as highly responsive to heating compared to the rest of the protein; by exposing hydrophobic residues to solvent and thereby triggering the overall STIM1 activation by aggregation of EF-SAM domains. Phe108 is one conserved residue that seems to play a crucial role in the process initiation. It might be hypothesized that STIM1 possesses a luminal thermal site (hEF), which triggers the downstream events of STIM1 dimer activation. A similar path (*via* EF-SAM dimerization) is used by the Ca^2+^ sensor (cEF), its close neighbor.

These computational results demonstrate the peculiar thermal sensitivity of EF-SAM domain and its potential role in STIM1 activation by temperature. Although there is not yet direct experimental evidence on hEF-hand involvement in STIM1 thermal sensing, several experimental findings indirectly support our proposed mechanism. Guldur *et al*. (41) showed using FRET measurements that extended unfolding is not required for EF-SAM dimerization and subsequent STIM1 activation. Enomoto *et al*. (73) found, using NMR measurements, that the most unstable residues at low Ca^2+^ concentration in EF-SAM coincide with those we found to be markedly unstable at moderately elevated temperatures in our simulations. The α3 helix is particularly noteworthy because it contains the F108 residue, which we proposed in our model as a potential thermal “hot-spot” residue responsible for EF-SAM dimerization upon temperature rise. They also showed that the SAM domain remains largely structured and α-helical in low Ca^2+^ concentration, consistent with our observations at moderately high temperatures. Schober *et al*. (55) and Sallinger *et al*. (56), using constitutively active STIM1 mutants selected from the cancer database and mutations associated with tubular aggregate myopathy (TAM) and live-cell recordings together with MD simulations, provided evidence that the hEF-hand and Phe108 play important roles in STIM1 activation by promoting dimerization while cEF and SAM retain their folded structure. If Ca^2+^ depletion triggers EF-SAM dimerization by inducing these structural changes, it is reasonable to hypothesize that similar structural changes induced by moderately elevated temperatures could also trigger dimerization and STIM1 activation, making it a compelling hypothesis to investigate in future experimental studies.

**Figure 9:**
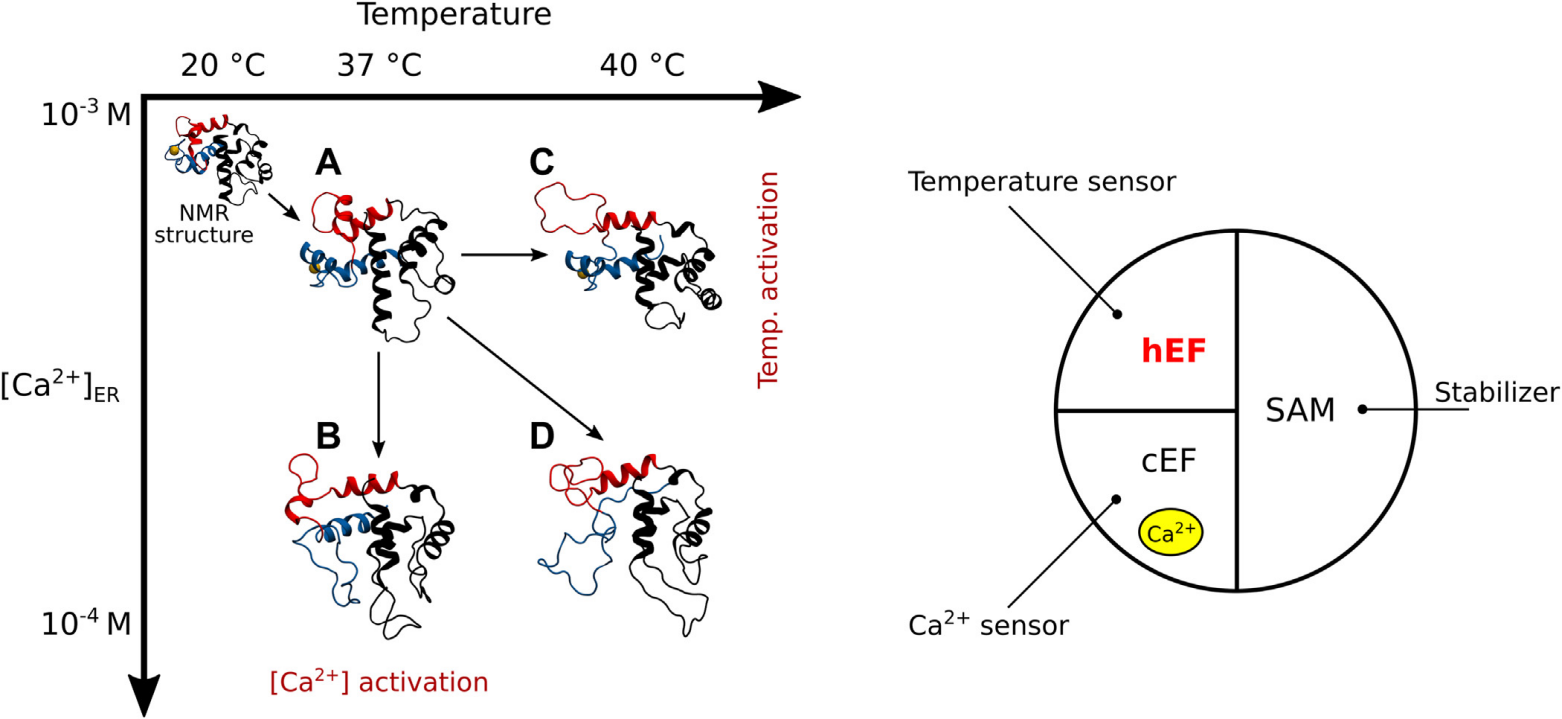
**STIM1 activation (*left*) may be triggered either by ER Ca**^**2+**^**store depletion or by temperature increase within several degrees.** EF-SAM calcium sensor already shows instabilities in hEF and SAM at 37 °C (*A*) compared to the structure at lower temperatures, while a slight decrease in ER [Ca^2+^] (*B*) or an increase in temperature (*C*), or both (*D*) can trigger a robust response. hEF (*red*), cEF (*blue*), SAM (*black*), Ca^2+^ (*yellow sphere*). The modular functional architecture of EF-SAM (*right*) includes a temperature sensor (hEF), a Ca^2+^ sensor (cEF), and a stabilizer domain (SAM).

Therefore, we propose a modular functional architecture for the multiple responsivities of the EF-SAM sensor, with three components (Fig. 9) (1): cEF-hand is a Ca^2+^ sensor (2); hEF-hand is a temperature sensor (3); SAM is a stabilizing domain. Such an architecture would explain the function of this complex and versatile sensor, which not only incorporates sensitivity for two distinct parameters but also allows reciprocal functional adjustments by allosteric influences between the two different detectors involved. Our data suggest a peculiar type of cooperation between these two detector elements of EF-SAM. Although not as a direct conclusion of our results, the functional impact of such roles of cEF and hEF, actually correlated, does open a new perspective upon the mechanism of SOCE: the temperature sensor seems to render the calcium sensor optimal at physiologically elevated temperatures, for the rapid activation of SOCE by a rather small decrease of reticular Ca^2+^ concentration.

## Conclusion

The present study brings together two different aspects of STIM1 sensing capabilities, temperature sensing, and calcium signaling, providing input for more extended analyses of their interplay. Understanding how STIM1 senses temperature is critical for gaining insight into the underlying mechanisms of temperature-dependent cellular processes essential in immune responses, fever response, gene expression, temperature sense, and metabolism. Proteins exist in a dynamic equilibrium between multiple conformations, and their population depends on factors such as temperature, pH, metal binding, and interaction partners. Our simulations showed that at 37 °C, the hEF subdomain of EF-SAM tends to occupy a partially unfolded conformation, which may expose the Phe108 residue to solvent. At lower temperatures, this tendency disappears, while at higher temperatures, it becomes more favorable. This suggests that slightly increasing the temperature above 37 °C may shift the equilibrium toward the unfolded state of hEF, which itself is sufficient to promote dimerization, as shown in previous experimental studies. Moreover, our findings also suggest that the temperature sensor renders the calcium sensor optimal at physiologically elevated temperatures for the rapid activation of SOCE by a decrease of reticular Ca^2+^ concentration, which has important implications for understanding the physiological role of STIM1 in temperature-dependent calcium signaling.

## Experimental procedures

### Modeling of the EF-SAM domain in solution

EF-SAM is the intraluminal N-terminal (N-t) topological domain of STIM1 (663 residues), with a length of 138 amino acid residues (positions 63–200, UniProtKB entry: Q13586). Here we used the NMR structure of a recombinant construct of human Ca^2+^-loaded EF-SAM domain (Protein Data Base (PDB) entry: 2K60) (20). The primary sequence of the recombinant protein (150 amino acid residues, including positions 58–201) differs from the natural variant of EF-SAM at its N-t end by the presence of an expression tag of six residues (positions −5 to 0 in the PDB entry). The engineered EF-SAM domain was shown to display independent folding, high stability in the presence of Ca^2+^, high fraction of per residue secondary structure, and high Ca^2+^ sensitivity, similar to the whole STIM1 protein (19, 20). Thus, we considered the 2K60 NMR structure as an appropriate model for the intraluminal EF-SAM Ca^2+^ sensor of STIM1. The first conformation in the 2K60 PDB entry was taken as the initial structure. We assumed constant standard protonation states for the ionizable groups at neutral pH. For the conventional MD simulations conditions included constant temperature (either 20 °C or 37 °C), constant pressure (1 atm), water as a solvent, and 150 mM KCl ionic strength. [Ca^2^] was not explicitly considered, low or high Ca^2+^ ionic strengths being modeled by either the absence or the presence of Ca^2+^ in the binding site.

### Systems preparation and equilibration

Much attention was paid to system construction and equilibration, to ensure that these steps do not introduce any unnatural conformational artifacts prior to production runs. For protein and ions description we used the OPLS (Optimized Potential for Liquid Simulations) all-atom force field (74). TIP4P was employed for water molecules (75). The initial EF-SAM structure including the bound Ca^2+^ (HOLO form) was solvated in a cubic simulation box with 7 nm edges, containing 10,165 water molecules. We added 41 K^+^ and 31 Cl^-^ ions to the simulation box, by replacing the adequate number of water molecules, in order to achieve a physiological salt concentration of 150 mM and an electrically neutral system. Everything was similarly done for the simulation of EF-SAM behavior after Ca^2+^ loss (APO form) but omitting Ca^2+^ from the 2K60 structure and compensating the respective charge loss by adding only 29 Cl^−^ ions to the solvent instead of 31 Cl^−^ ions. The systems were energy minimized, followed by a suite of consecutive equilibration MD stages in which the restraints applied on subgroups of protein atoms were gradually relaxed (first all heavy atoms, then backbone atoms, and in the end only C-α atoms). In these equilibration simulations, the water molecules together with K^+^ and Cl^−^ ions were free to move and adapt to the local protein microenvironment. The total equilibration period was 14 ns, enough for the solvent to intimately equilibrate with the protein surface.

### Conventional MD simulations of EF-SAM

Four sets of MD production runs were performed under different conditions, namely: HOLO-37 (Ca^2+^, 37 °C), APO-37 (no Ca^2+^, 37 °C), HOLO-20 (Ca^2+^, 20 °C), and APO-20 (no Ca^2+^, 20 °C). Each set contained 12 independent simulations thermalized with randomly generated velocities from a Maxwell distribution at the specified temperature. One simulation from each set was continued up to 1.8 μs. Production simulations were performed without any atomic position restraints. All bond lengths were constrained *via* the LINCS (linear constraint solver) algorithm (76) to the equilibrium distances provided by the OPLS force field. Virtual sites were used to achieve a 5 fs integration time step. We used the “constraint free approach” to describe the metal center (77) and closely monitored Ca^2+^ persistence within the binding site. All production runs were done in the isothermal-isobaric ensemble (NPT—constant number of particles, pressure, and temperature). Pressure was constrained, by coupling the simulation box to an external Berendsen barostat, at 1 atm and a relaxation time constant of τ = 0.5 ps. For thermal coupling, we followed the common scheme with two different thermostats, one coupled to the solvent (including ions) and the other coupled to the simulated protein (78). Thermostats were set to the reference temperatures of 310 K or 293 K, depending on the simulation type. The "v-rescale" thermostat was used, as implemented in GROMACS 4.6. This thermostat is very efficient, does not suffer from ergodicity problems, and minimally perturbs the simulated systems (79). The short-range non-bonded interactions were cut off at 10 Å. For long-range electrostatic interactions, we used the Particle Mesh Ewald (PME) method (80), with a grid spacing of 0.12 nm and cubic interpolation of electrostatic forces. Snapshots of the protein and ion positions were saved every 4 ps for subsequent analysis using GROMACS 4.6 (81).

### Analysis of the MD trajectories

To quantify the conformational changes of the protein following Ca^2+^ un-binding and structure conservation of the Ca^2+^ bound form we analyzed the root-mean-square deviations (RMSD) and root-mean-square fluctuations (RMSF) of the protein backbone. These were computed using the g_rmsf and g_rms utilities from the GROMACS 4.6 suite, after the removal of protein overall rotation and translation by a prior fitting of the trajectory snapshots to the starting structure. The fitting involved only the C-α atoms of the residues Ser64 to Leu199, as the N-t (Gly(-5) to Leu63) is not well-defined in the NMR structure and highly fluctuates during simulations. RMSFs were averaged per amino acid residue. Conformational changes were also monitored by radius of gyration (R_g_) analysis using the g_gyrate program in GROMACS suite. Residue contact maps were evaluated by computing the inter-residue distance matrices consisting of the smallest distances between each residue pair in selected subsets. Contact maps were evaluated using the g_mdmat program for seven frames equally distributed at 300 ns intervals along the μs range simulations. Secondary structure evolution during the simulations was monitored using the DSSP program (82) (do_dssp tool in GROMACS 4.6) on snapshots equally distributed at 1 ns time intervals. Visual inspection of the trajectories and numerical data analysis was done using in-lab written scripts and VMD (http://www.ks.uiuc.edu/Research/vmd/) (83).

### Replica exchange molecular dynamics simulations

Solute tempering (REST2) (84) simulations were performed with the PLUMED (85) plugin for GROMACS on the Beskow supercomputer at PDC Center for High Performance Computing at KTH Stockholm, Sweden. REST2 simulations were performed with two reference minimum temperatures of 15 °C and 20 °C respectively, using the CHARMM36m force field (86), which is suitable for both folded and intrinsically disordered proteins. Force field was intentionally changed in REST2 simulations to test the robustness of the results and ensure they reflect the physics of the system rather than specific force field influences. Each REST2 simulation used 30 replicas, with λ between 1 and 0.6. Within each REST2 simulation, each replica evolved at the same reference temperature. The length of REST2 simulations was 280 ns for each replica, and only the last 80 ns were used for analysis. The thermal stability curves were constructed based on the law of corresponding states. The effective temperature scale of the replicas was obtained by converting the λ-scale, as previously described by Stirnemann *et al*. (61). The α-helical content of the entire EF-SAM was used to define the reaction coordinate for the thermal stability profile construction (87). To dissect the individual contribution of cEF, hEF and SAM domains to the overall protein unfolding, we must adopt a consistent definition of the folded state for each of them (Fig. S8). Usual RMSD or the α-helical content alone were not suitable candidates as quantitative parameters for cEF and hEF, due to the following reasons. Visual inspection of the conformations in different replicas revealed that EF-hands for example may show a total lack of helical structure on one of their α-helices while still having a low RMSD. Conversely, other conformations displayed high helical content while having a high RMSD. However, both situations describe an unfolded state. Therefore, a comprehensive set of requirements has been established for cEF and hEF to be considered folded. For hEF these are: (i) the α3 and α4 helices maintain their helical structure; (ii) residue Phe108 is buried in between α3, α4 and α10 helices (this assures also that the α3 helix is anchored on the α10 of SAM). For cEF the criteria are: (i) the α1 and α2 helices maintain their helical structure; (ii) the Ca^2+^ binding loop is not expanded; (iii) SAM’s α10 is anchored in between α1 and α2. For each replica, a cluster analysis was performed using GROMACS software suite. The median conformation of each cluster was analyzed with respect to the above criteria. If all the requirements were fulfilled, the cluster was considered to represent a folded conformation, and the number of its members was added to the total number of folded conformations. The individual thermal stability curves for cEF and hEF were represented as the percentage of unfolded conformations, from the total number of conformations, *versus* the effective temperature. For SAM, which was much more stable, the α-helical content proved to be enough for constructing the thermal unfolding profiles (87).

## Data availability

The datasets/trajectories and computer scripts used in this study are available upon request to the corresponding author.

## Supporting information

This article contains supporting information (28, 88, 89, 90, 91, 92, 93, 94).

## Conflict of interest

The authors declare that they have no conflicts of interest with the contents of this article.
