# Supplementary material for: Molecular dynamics simulations reveal the hidden EF-hand of EF-SAM as a possible key thermal sensor for STIM1 activation by temperature
Source: J Biol Chem. 2023 Jun 27;299(8):104970. doi: 10.1016/j.jbc.2023.104970 (PMC10400917; doi:10.1016/j.jbc.2023.104970)
Supplement: Supporting information [file mmc1.docx]

**SUPPORTING INFORMATION**

**Molecular dynamics simulations reveal the hidden EF-hand of EF-SAM as a possible key thermal sensor for STIM1 activation by temperature.**

Andrei Neamtu^1,2^, Dragomir N. Serban^1^*, Greg J. Barritt^3^, Dragos Lucian Isac^2^, Tudor Vasiliu^2^, Aatto Laaksonen^4,5,6^, Ionela Lacramioara Serban^1^

^1^Department of Physiology, “Grigore T. Popa” University of Medicine and Pharmacy, Universitatii Str. No. 16, Iasi 700115, Romania

^2^Center of Advanced Research in Bionanocojugates and Biopolymers, “Petru Poni” Institute of Macromolecular Chemistry Iasi, Grigore Ghica Voda Al. No. 41A, Iasi 700487, Romania

^3^Discipline of Medical Biochemistry, College of Medicine and Public Health, Flinders University, GPO Box 2100, Adelaide SA 5001, Australia

^4^Department of Materials and Environmental Chemistry, Arrhenius Laboratory, Stockholm University, 106 91 Stockholm, Sweden

^5^Centre of Advanced Research in Bionanoconjugates and Biopolymers, Petru Poni Institute of Macromolecular Chemistry, Aleea Grigore Ghica-Voda, 41A, 700487 Iasi, ROMANIA.

^6^State Key Laboratory of Materials-Oriented and Chemical Engineering, Nanjing Tech University, Nanjing, 210009, P. R. China.


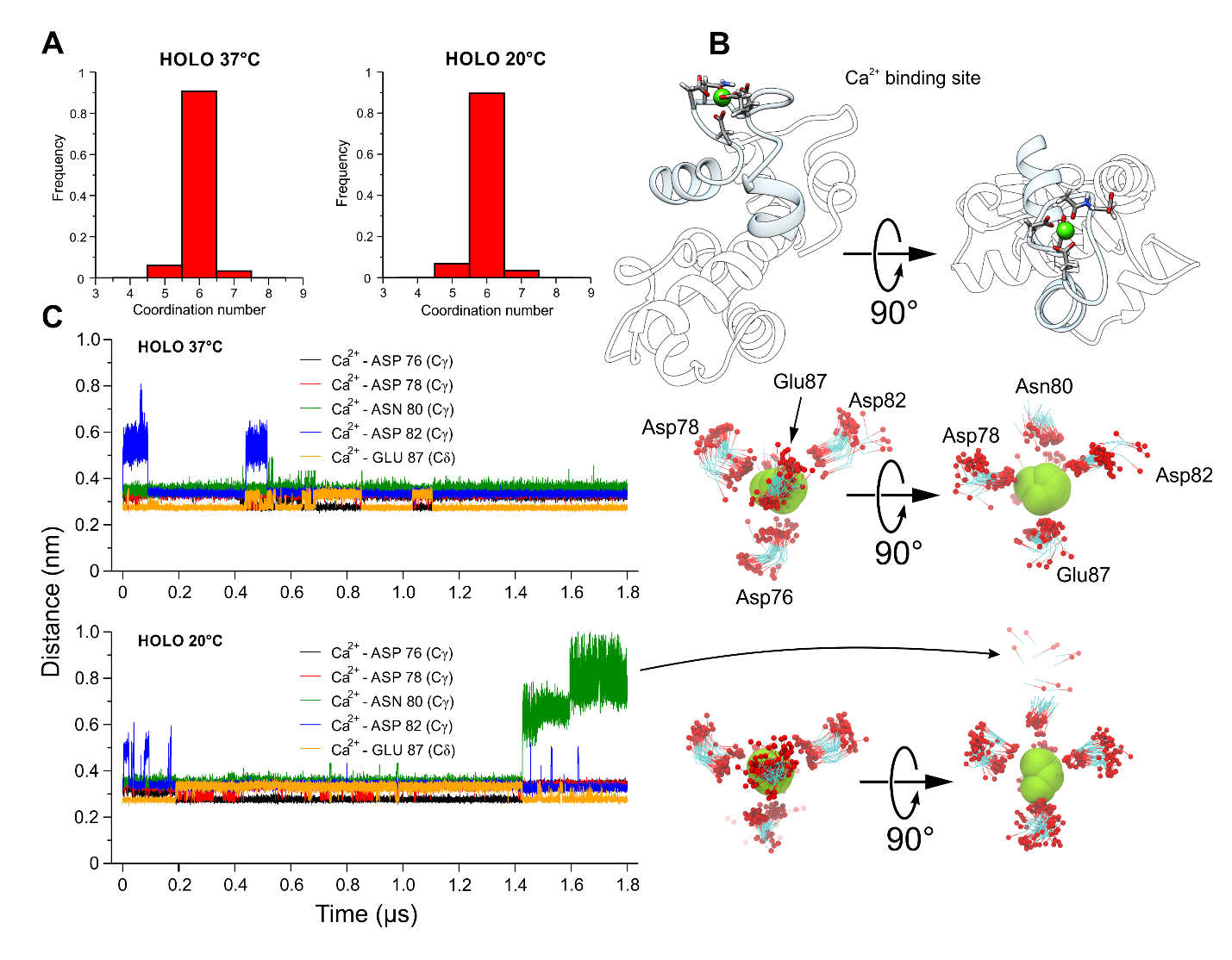


**Fig. S1.** Chelated Ca^2+^ stability in extended microsecond simulations. (A) Coordination number of Ca^2+^ by canonical EF-hand (cEF) liganding residues. A contact was considered to exist between the protein residues and the cation when the inter-nuclear distance between the residue atoms and the cation was smaller than 3 Å. (B) (above) Ca^2+^ binding site geometry within the protein. Binding site liganding residues were represented as sticks and the cation as a green van der Waals sphere; (below) detail of the binding site residues and the cation as successive MD snapshots. (C) Distances between Ca^2+^ and different carbon atoms of the chelating residues (denoted in parenthesis). Due to the long-time scale of the extended simulations, one major concern over the accuracy of HOLO simulations was Ca^2+^ stability inside the binding site. A possible Ca^2+^ separation from the protein would have had the undesirable effect of converting the HOLO form to the APO form. Distances between Ca^2+^ and different representative carbon atoms (Asp76(Cγ), Asp78(Cγ), Asn80(Cγ), Asp82(Cγ), and Glu87(Cδ)) were monitored. The pentagonal bipyramidal geometry of the site is well preserved, as shown by different snapshots of the MD trajectories (Fig. S1 - B). As expected, the invariant Glu87 at position '12' provided two oxygen atoms for Ca^2+^ binding (bidentate ligand) at both temperatures studied. The coordination number, defined as the number of side chain oxygen atoms of the binding site residues closer than 3Å from Ca^2+^, had a mean value of 6 (Fig. S1 - A), with a narrow distribution centered on this value, in agreement with a pentagonal bipyramidal geometry with a water molecule at position ‘9’. Simultaneously, the Asp82(7,-Y) side chain became one of the coordinating partners for Ca^2+^ (Fig. S1 - C).


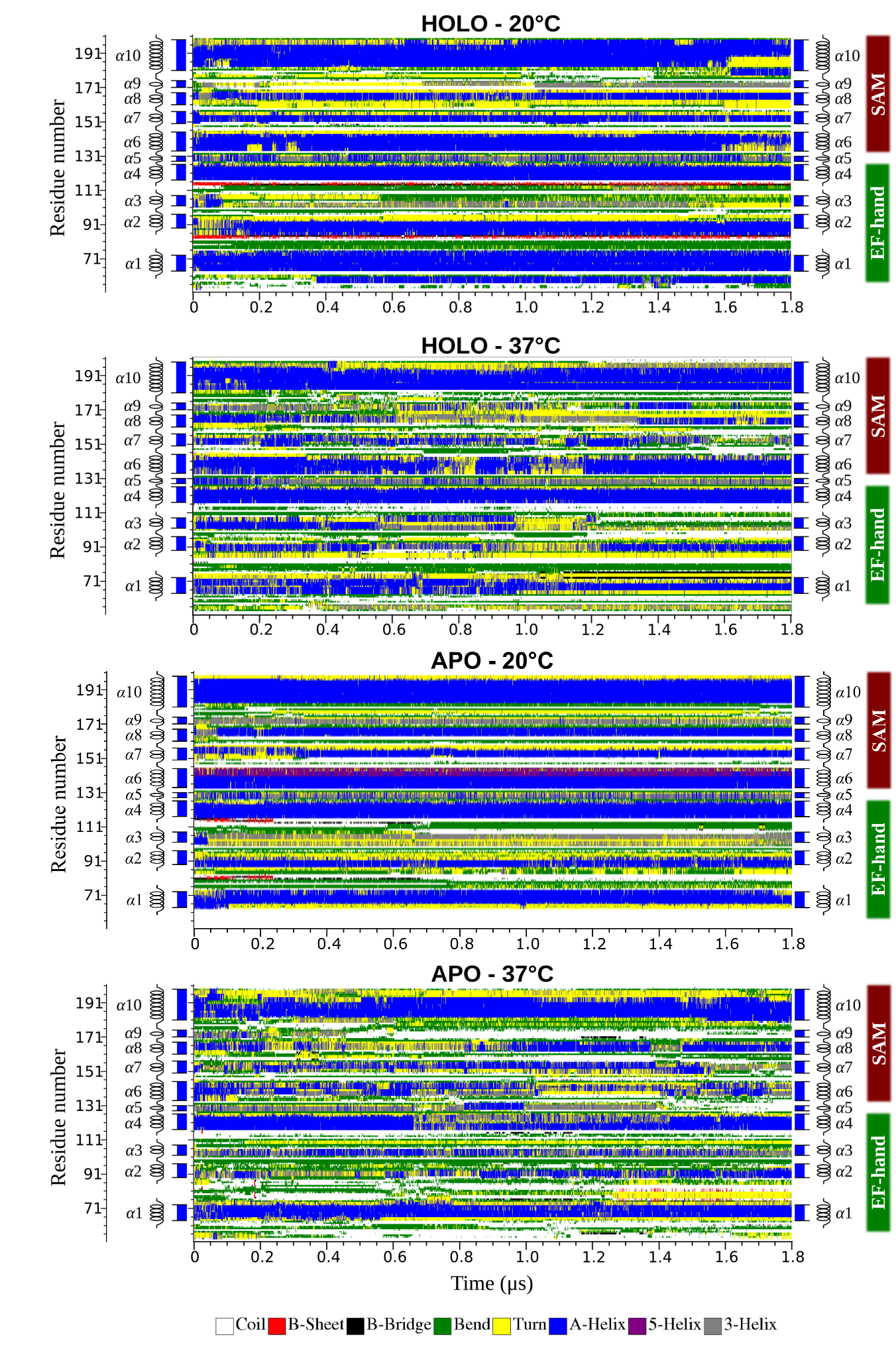


**Fig. S2.** Secondary structure evolution during the molecular dynamics simulations of the EF-SAM domain. In the HOLO-20 simulation the α9 and α3 helices showed the lowest stability, making transitions between turns and 3_10_-helices, a feature that is commonly encountered in short helices in proteins (Armen, 2003) The α5 helix, that links the EF-hand subdomain to SAM, remains mainly helical, making 3_10_- to α-helix transitions. The α1 helix becomes somehow more extended, to include also the Leu74 and Met75 residues. The β1 and β2 strands, that make up the sheet specific for paired EF-hand motifs, were well preserved during the entire HOLO-20 simulation.


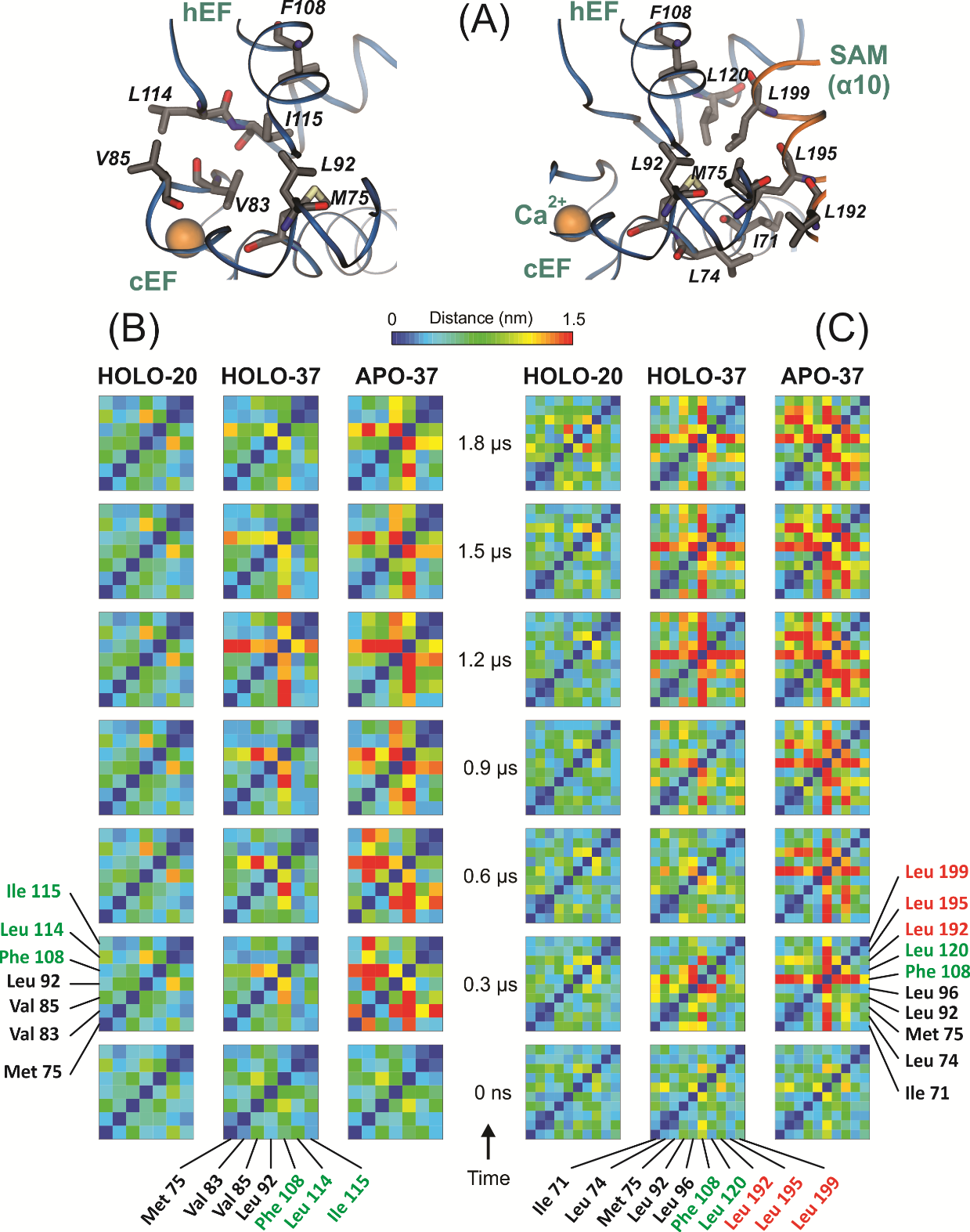
**Fig. S3.** (A) Hydrophobic interaction interfaces between the hidden and canonical EF-hands (hEF-cEF) (left) and between the EF-hand pair and α10 helix of SAM (EF-pair - SAM) (right), together with their representative residues. (B) Hydrophobic contact maps for selected residues in cEF-hEF. (C) EF-pair - SAM interfaces. The hydrophobic packing between the two EF-hands and between the EF-hand pair and SAM is highly affected by Ca^2+^ removal (indicated by increased frequency of light green-yellow-red colors appearance in contact maps). At the cEF-hEF interface (Fig. S3 A, B) the minimum distances between Phe108 and residues Leu75, Val83, Val85 and Leu92 become highly increased due to Phe108 solvent exposure. Other more direct contacts are lost, including Val83 (β1-cEF) – Ile115 (β2-hEF) and Leu92 (α2-cEF) – Ile115 (β2-hEF), both of which directly stabilize EF-hands pairing. The direct contact between Phe108 (α3-hEF) and Ile115 (β2-hEF) is also compromised, leading to hEF destabilization. Likewise, within the EF-hand pair - α10 (SAM) interface (Fig. S3 A, C), the overall hydrophobic network becomes highly destabilized. The anchoring residues on α10 give rise to extended hydrophobic interactions in the folded state, as follows. Leu199 interacts with Met75 (α1-cEF), Leu92 (α2-cEF), Leu96 (α2-cEF), Phe108 (α3-hEF), and Leu120 (α4-hEF). This large hydrophobic interface becomes widely undermined at Leu199 contacts with Leu92-Phe108-Met75. A second EF-SAM hydrophobic anchor is provided by Leu195, which closely contacts Ile71 (α1-cEF), Leu74 (α1-cEF), and Met75 (α1-cEF). Two of these contacts were lost in APO-37: Leu195 to Leu74 and Leu195 to Met75. The third anchoring residue of α10 helix, Leu192, has hydrophobic interactions with Leu96 (α2-cEF) in the folded state, but the close contact is lost in the APO state. A certain degree of hydrophobic core destabilization is also present at 37 °C in the presence of Ca^2+^ (HOLO-37), though to a much lesser extent. For HOLO-37, the altered contacts were only: Leu199 to Phe108-Leu96-Leu92 and Leu195 to Leu74. Still, the interface between the two EF-hands is preserved. In contrast, at 20 °C in the presence of Ca^2+^ (HOLO-20), the hydrophobic core of EF-SAM shows high stability and increased hydrophobic network conservation over the μs time scale. The two red squares at 1.8 μs for HOLO-20 are due to an increase in the distance between two un-related, distant residues: Leu120 and Leu96.


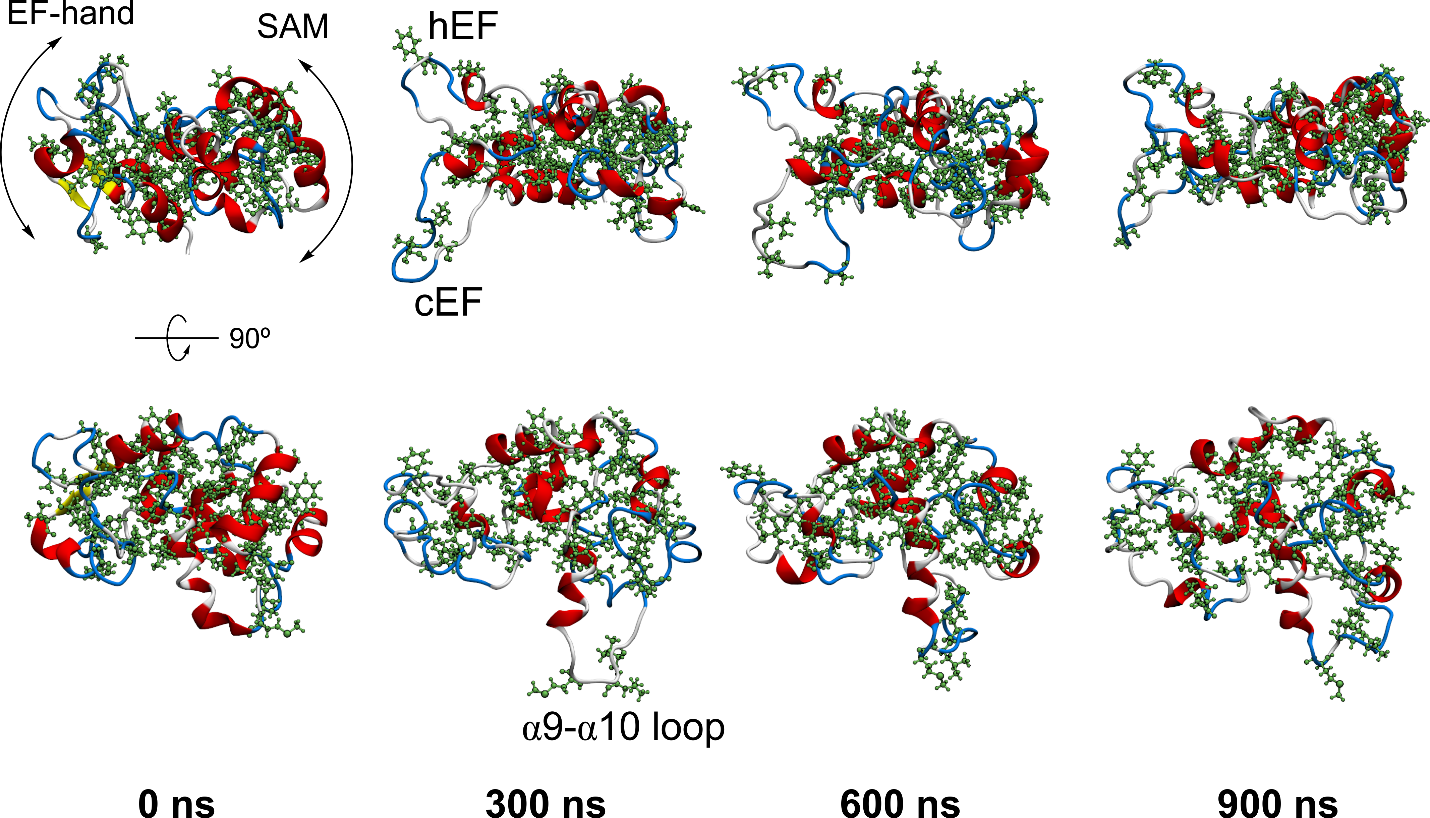


**Fig. S4.** Diagram depicting the hydrophobic residues (green ball and stick) solvent exposure during the first 900 ns of the molecular dynamics simulations of the EF-SAM domain of STIM1 without Ca^2+^ at 37 °C.


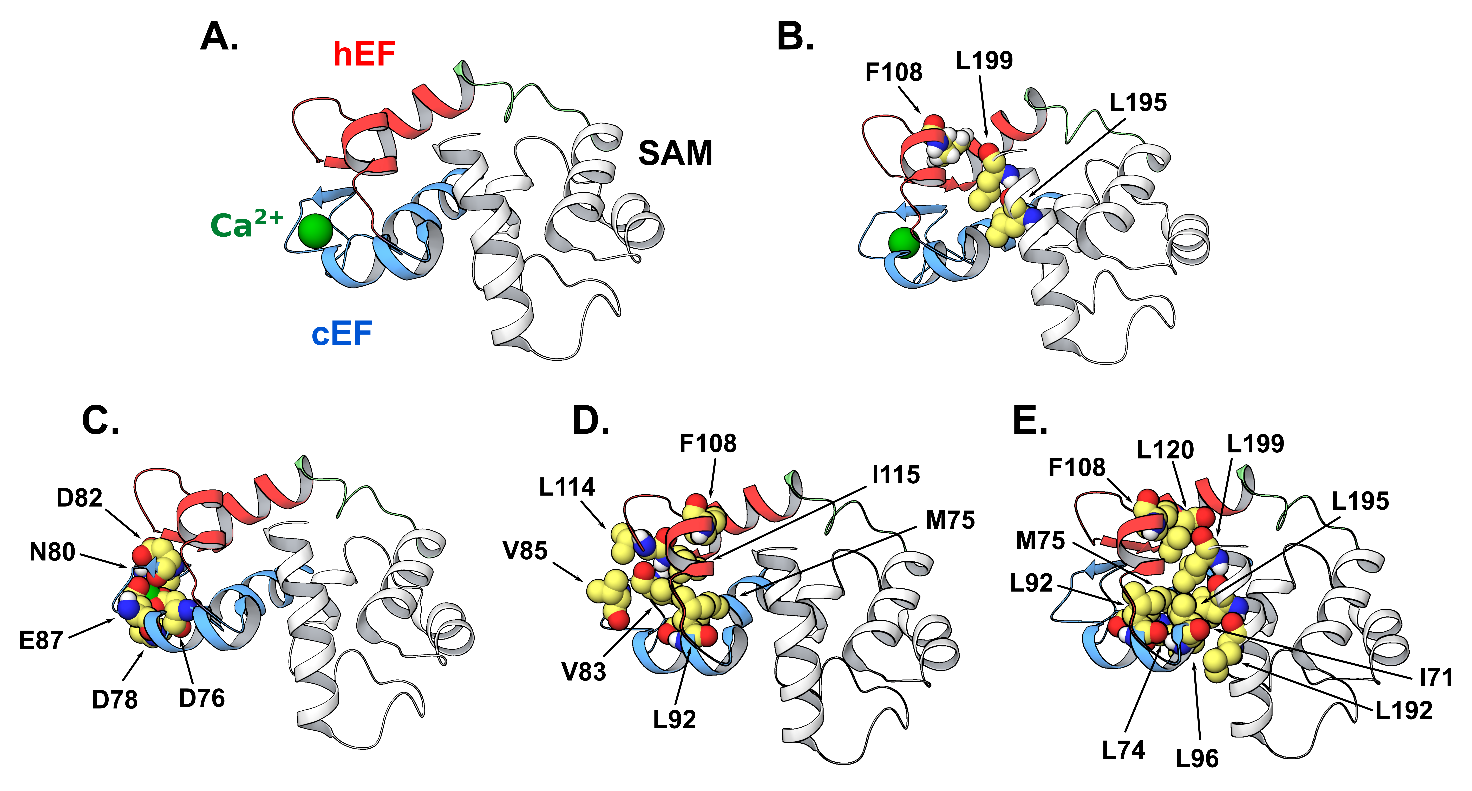


Fig. S5 Fig. S5. The spatial arrangement of the residues relevant for EF-SAM stability. (A) The general structure of the EF-SAM domain with color-coded cEF, hEF, and SAM subdomains. (B) The spatial relationship between Phe108 on hEF and the α10 helix on SAM, highlighting residues Leu199 and Leu195. (C) Residues involved in the Ca^2+^ binding site. (D) Hydrophobic interaction interface between the hidden and canonical EF-hands (hEF-cEF). (E) Hydrophobic interaction interface between the EF-hand pair and the α10 helix of SAM (EF-pair - SAM).

**
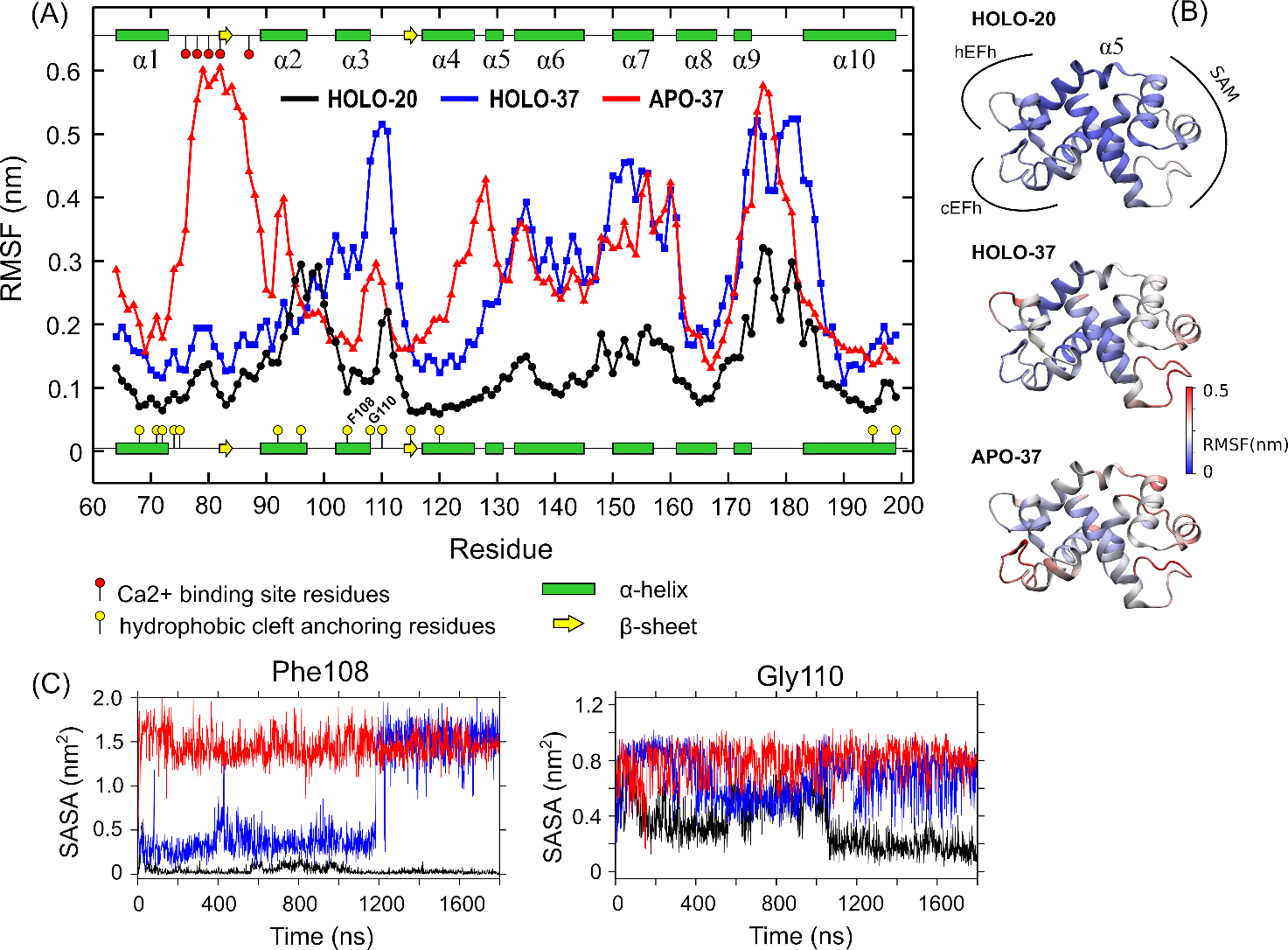
**

**Fig. S6.** Molecular dynamics (MD) simulations of the EF-SAM domain of stromal interaction molecule 1 (STIM1), with Ca^2+^ bound (HOLO) or not (APO); at 37 ^o^C (HOLO-37 or APO-37) or at 20 ^o^C (APO-20). (A) Root mean square fluctuations (RMSF) of atomic positions averaged over each residue; Ca^2+^ binding site residues and hydrophobic cleft anchoring residues were highlighted by red and yellow "ball and stick" glyphs. (B) Ribbon diagrams on the right show RMSF mapped to the protein backbone for an easier identification of RMSF variation in the global context of the protein structure. (C) Solvent accessible surface area of Phe108 sidechain and Gly110 backbone for HOLO-20 (black), HOLO-37 (blue) and APO-37 (orange); the sequence Gly(-5) to Lys62 was not included in the analysis (see methods).

Analysis of the backbone RMSFs for APO-37 and HOLO-37 revealed significant differences compared to HOLO-20. Four regions of enhanced flexibility have been identified: (1) C-t of α3 plus α3-β2 loop (Thr107 to Gly110) (hEF); (2) C-t of α4 up to α8 (Ala123 to Gly161) (SAM); (3) α9 plus α9-α10 loop (Thr172 to Thr182) (SAM); (4) α1-α2 loop plus α2 (Leu74 to Glu94) (cEF). The fourth one matches the Ca^2+^ binding site and its markedly increased RMSF only for the APO form suggests a strong destabilization upon Ca^2+^ unbinding. Previous MD studies also suggested Ca^2+^ binding site conformational instability in the APO state, though the simulations were far shorter (50 ns) (51, 52).

The full exposure of the Phe108 side chain and of the Gly110 backbone towards the solvent in the APO form indicates that the geometrical rearrangements of the α3-β2 loop were appreciable in this case. Given the space vicinity between the α1-β1 and α3-β2 loops, we assume that the peaks relative to these two regions are coupled, the fluctuations arising from the disruption of their interaction interface. The most stable regions in the EF-hand pair corresponded to α1-to-α4 contact residues (i.e. α1 and N-t of α4, residues 64 to 73 and 117 to 122 respectively).

The α1 helix is highly stable for both APO and HOLO, as shown by the conserved α-helical structure in the secondary structure plots for all four extended simulations (Fig. S2). The α4 helix is also stable in APO and HOLO varieties, with a temporary loss of structure and transitions to a 3_10_ helix at ~600 ns in the case of APO-37. Interestingly, in simulations performed at 37 °C, in the presence as well as in the absence of Ca^2+^, the small *β*-sheet between the EF-hands is not present. Without any experimental structural data for the EF-SAM domain at physiological temperatures, it is difficult to clarify if this is really a feature present only at lower temperatures or it is a limitation imposed by the incomplete phase space coverage by computational means. However, it is accepted that the two hydrogen bonds that stabilize the β-sheet display significant fluctuations in the APO compared to the HOLO forms of other EF-hand pairs (Marchand, 1998). Moreover, while β-sheets are important for the structural integrity and/or cooperativity of many EF-hand pairs, it is not indispensable for others, where missing, e.g. sarcoplasmic Ca^2+^-binding protein from *Amphioxus*(88).

In the SAM sub-domain, high flexibility/disorder is detectable in the same regions for both HOLO-37 and APO-37. The α6, α7 and α8 helices display rich helical content, but with frequent α to 3_10_ interconversions (Fig. S2). The α9 loses its helical structure and together with the α9-α10 loop shows significant disorder. This was also suggested by a previous replica exchange MD simulation study(51). There, the increased α9-α10 loop solvent exposure was considered as an indicator of an intermediate transition state on the APO unfolding pathway, as it exposes hydrophobic residues that can initiate EF-SAM dimerization.


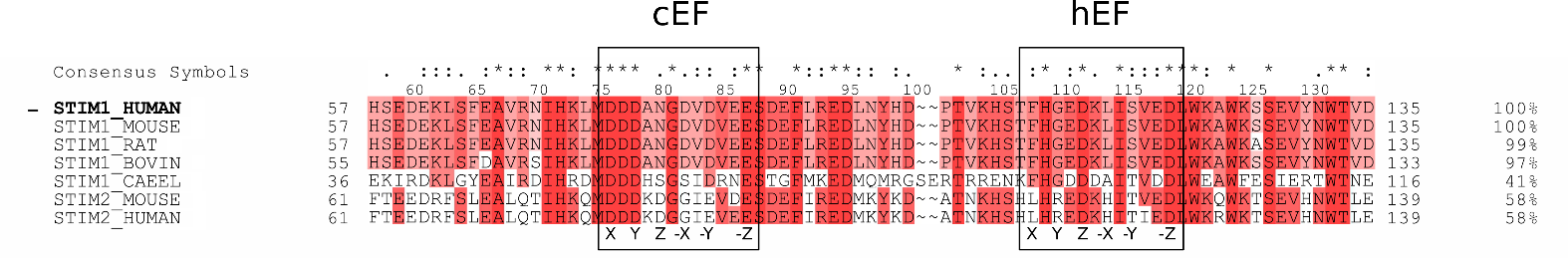


**Fig. S7.** Sequence alignments of the EF-hand domain of stromal interaction molecule (STIM) variants (i.e. various STIM1 and STIM2 proteins). Canonical (cEF) and hidden (hEF) EF-hands are highlighted with the usual notation for the residue positions and orientations. Red background tints are used to express the aligned residue identity. On the right side, the entire sequence percentage identity of each STIM variant is compared to the human STIM1. UniProt access codes: STIM1_HUMAN (Q13586)(28), STIM1_MOUSE (P70302)(89), STIM1_RAT (P84903)(90), STIM1_BOVIN (Q58CP9)(91), STIM1_CAEEL *(Caenorhabditis elegans)* (G5EF60)(92), STIM2_MOUSE (P83093)(93), STIM2_HUMAN (Q9P246)(94).


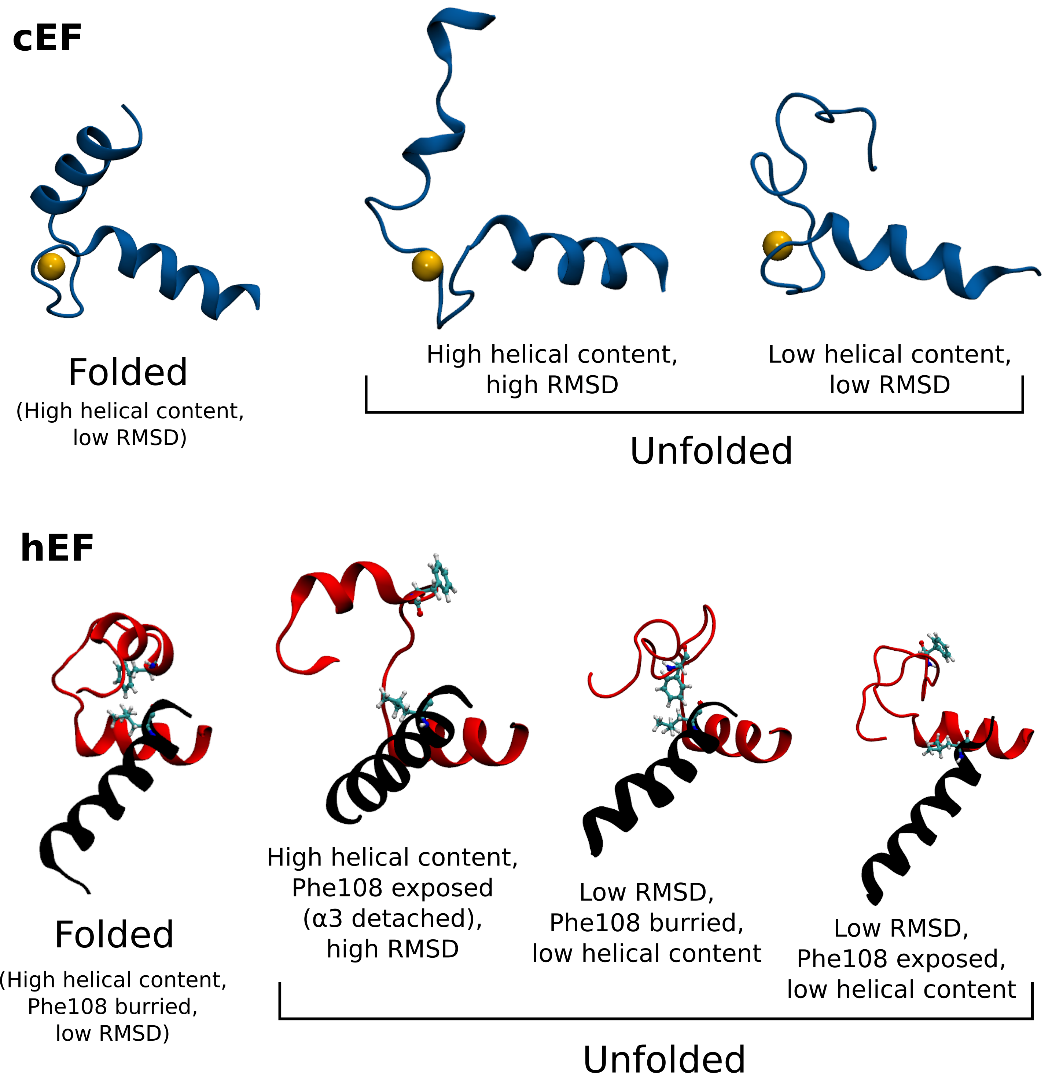


**Fig. S8.** Definition of the folded and unfolded states of cEF and hEF.
